# Natural Products from Leaves of the Ancient Iranian Medicinal Plant *Echium amoenum* Fisch. & C. A. Mey.

**DOI:** 10.3390/molecules28010385

**Published:** 2023-01-02

**Authors:** Mehdi Jafari Jamnani, Bjarte Holmelid, Anni Vedeler, Hoda Houshiar Parsian, Heidi Lie Andersen, Torgils Fossen

**Affiliations:** 1 Department of Chemistry and Centre for Pharmacy, University of Bergen, Allégt. 41, 5007 Bergen, Norway; 2 Department of Biomedicine, University of Bergen, 5009 Bergen, Norway; 3 University Gardens, University of Bergen, Allégt. 41, 5007 Bergen, Norway

**Keywords:** *Echium amoenum*, leaves, polyphenols, caffeoyl derivatives, 2D NMR

## Abstract

For several millennia, leaves of *Echium amoenum* Fisch. & C. A. Mey., an important Iranian medicinal plant with nutritional value as nutraceutical, have been used as tea for the treatment of several conditions, including inflammation. The nutritional value of intake of *E. amoenum* tea has mainly been correlated to its rich content of mainly water-soluble antioxidants. Although the entire plant is utilized, only natural products of the flowers have previously been thoroughly investigated. The rare natural products bis(3-(3,4-dihydroxyphenyl)-1-methoxy-1-oxopropan-2-yl)-1-(3,4-dihydroxyphenyl)-6,7-dihydroxy-1,2-dihydronaphthalene-2,3-dicarboxylate, 4-Oxy-(*E*)-caffeoyl-2,3-dihydroxybutanoic acid methyl ester and 4-Oxy-(*Z*)-caffeoyl-2,3-dihydroxybutanoic acid methyl ester, in addition to the widely distributed compounds rosmarinic acid methyl ester and (*E*)-caffeic acid, were purified and characterized from leaves of *Echium amoenum*. The structures were determined by a combination of several 2D NMR spectroscopic techniques, circular dichroism spectroscopy and high-resolution mass spectrometry. The fact that bis(3-(3,4-dihydroxyphenyl)-1-methoxy-1-oxopropan-2-yl)-1-(3,4-dihydroxyphenyl)-6,7-dihydroxy-1,2-dihydronaphthalene-2,3-dicarboxylate belongs to a rare group of natural products which have previously been patented for their significant anti-inflammatory activity may rationalize the traditional treatment of inflammations with *E. amoenum*.

## 1. Introduction

Traditional medicine is deeply rooted in Iranian culture and history. Avicenna (980–1037), the great 11th century Persian scientist and philosopher of the Middle Ages, provided comprehensive information on the preparations of various medicinal plants in his epoch-making encyclopedia *Canon of Medicine* (Qanun), which has been used as a textbook in medicine in the world’s most renowned universities for more than 500 years. It provides extensive information on various drug preparations from a multitude of medicinal plants, which today are frequently used in Iranian traditional medicine [1]. Because of the long history of utilizing medicinal plants, in addition to the variations in climate and geographical conditions, the flora in Iran is characterized by high biodiversity and is home to a wide variety of species of the plant kingdom. At least 1000 different species of medicinal plants have been identified [1].

**Figure 1:**
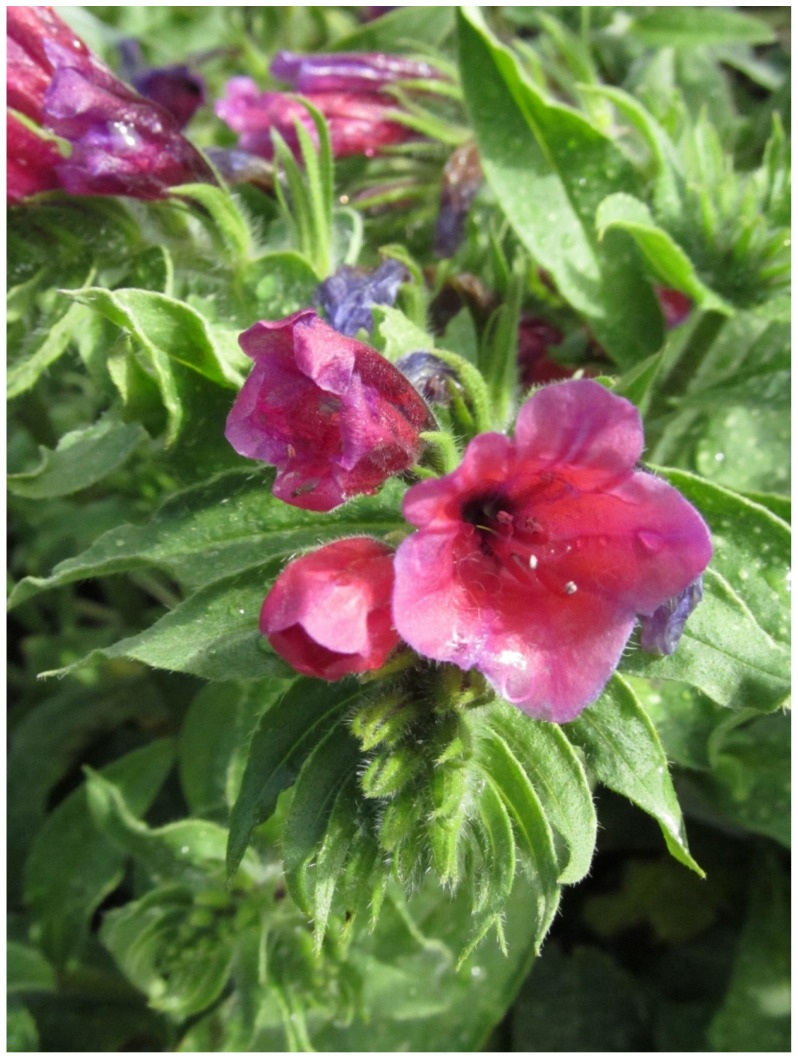
*Echium amoenum* cultivated in the Botanical Garden of the University of Bergen, Norway, photographed Friday 8 August 2014, at 10:30 a.m. Photo: Torgils Fossen.

*Echium amoenum* Fisch & C.A. Mey. (Figure 1) belongs to the family Boraginaceae and is a perennial Iranian domestic plant which grows wild in the mountains along a narrow strip of land in Northern Iran and the Caucasus [2]. *E. amoenum* is a valuable medicinal herb which has been used in the treatment of inflammatory and infectious diseases for more than a millennium. *E. amoenum* is also used as a sedative in the traditional treatment of stress and anxiety. In Iran, it is a common tradition to brew portions of dried flowers of *E. amoenum* and drink it as tea. As far back as a millennium ago, Avicenna described the use of *E. amoenum* in various pharmaceutical preparations for the treatment of infectious and inflammatory diseases in his medical encyclopedia *Canon of Medicine*. Among other things, he describes a pharmaceutical formulation of *E. amoenum*’s ash as an effective remedy in the treatment of cold sores and oral infections. In *Makhzan-al-Adwiya*, written by Khorasani, also one of the great Middle Ages Iranian scientists and physicians, *E. amoenum* was described as a remedy for coughs, sore throats, colds, pneumonia and dyspnea [1].

Despite Avicenna’s detailed descriptions of approaches for more research on this medicinal plant, it was not until recent years that *E. amoenum* was investigated in a number of scientific studies [3]. Even today, despite the great conviction and acceptance of the Iranian population of *E. amoenum*’s versatile and unique properties in the treatment of several different diseases, only a few thorough studies have been performed on this widely used medicinal plant. There are three different species of the genus *Echium* (Boraginaceae) in Iran, including *E. amoenum, E. russicum* J.F.Gmel. (now *Pontechium maculatum* L.)*,* the widespread *E. italicum* L. and the very rare and endemic *E. khuzistanicum* Mozaff. [4]. Only *E. amoenum* has been found to possess valuable medical applications [5]. In Iran, dried violet-red petals of *E. amoenum*, as well as the leaves, are used in a variety of medical contexts as anti-inflammatory, antibacterial, antioxidant, immunomodulatory, analgesic, anxiolytic, water-retardant and sedative herbal remedies. The best time to harvest the flowers is mid-May and early June, when the secondary metabolites of *E. amoenum* are assumed to be at their highest quantitative levels, thus accumulating the maximum amount of active substances in the flowers [6]. Various phytochemical studies conducted on *E. amoenum’s* petals and seeds confirm the presence of various natural products such as antocyanins, flavonoids, saponins, unsaturated terpenoids, pyrrolizidine alkaloids, sterols and various fatty acids with significant amounts of γ-linolenic acid [5]. Even the presence of some essential oils in smaller amounts, with the bright yellow δ-cadinene as the major compound, is demonstrated in *E. amoenum* [2].

The nutritional value of intake of *E. amoenum* tea has mainly been correlated to the fact that the plant is a rich source of mainly water-soluble antioxidants [7]. In view of the large, frequent consumption of *E. amoenum* as an effective natural remedy among the population of Iran, it is evident that more research is required to solve many unanswered questions about this medicinal plant. An important aspect thereof is the isolation and characterization of new natural products from *E. amoenum*, with relevant biological activity in relation to those indications for which the plant is utilized, which can pave the way for the development of new drugs from this medicinal plant. Although the leaves of *E. amoenum* have been used for similar therapeutic applications to that of the flowers, limited knowledge is available about the chemical constituents of the leaves in the current literature. In this paper, we report the first identification of polyphenolic compounds and their structure from the leaves of *E. amoenum*.

## 2. Results and Discussion

**Figure 2:**
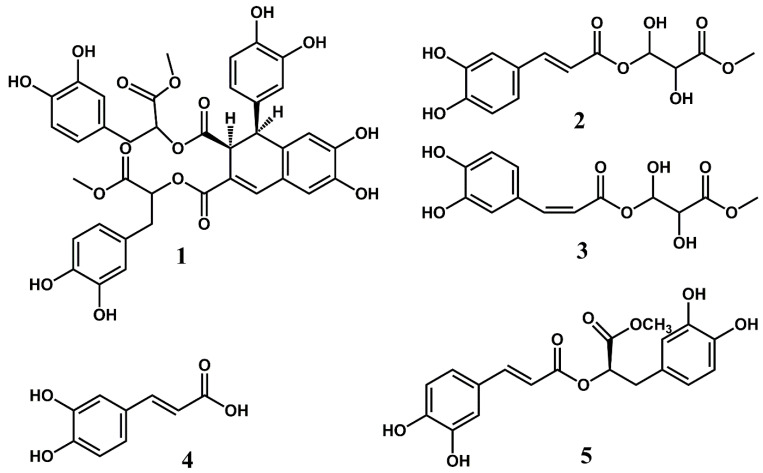
The molecular structures of compounds **1**–**5** characterized from leaves of *Echium amoenum*.

The UV spectrum of compound **1** recorded on-line during HPLC analysis exhibited λ_max_ values at 231 nm, 255 nm, 318 nm (sh) and 344 nm, which is in accordance with an aromatic compound with a relatively extensive chromophore. The 1D and 2D NMR spectra of **1** (Figures S8–S14) showed the presence of a molecule consisting of four condensed caffeic acids (Figure 2). The downfield region of the 1D ^1^H NMR spectrum of compound **1** showed eight phenolic hydroxyl protons at δ 9.45 (7-OH), δ 9.09 (6-OH), δ 8.80 (3‴-OH), δ 8.75 (3″-OH), δ 8.74 (4‴-OH), δ 8.730 (3′-OH), δ 8.726 (4″-OH) and δ 8.69 (4′-OH), an ABX system at δ 6.26 (d 2.3 Hz, H2′), δ 6.21 (dd 8.2, 2.3 Hz, H6′) and δ 6.56 (d 8.2 Hz, H5′), a second ABX system at δ 6.57 (d 2.1 Hz, H2′’), δ 6.42 (dd 8.0, 2.1 Hz, H6′’) and δ 6.61 (d 8.0 Hz, H5″), a third ABX system at δ 6.60 (d 2.1 Hz, H2‴), δ 6.43 (dd 8.0, 2.1 Hz, H6‴) and δ 6.63 (d 8.0 Hz, H5‴), two aromatic protons at δ 6.86 (s, H5) and δ 6.39 (s, H8) and an alkene proton at δ 7.56 (s, H4). In the aliphatic region of the 1D ^1^H NMR spectrum and the 2D ^1^H-^1^H COSY and the ^1^H-^13^C HSQC-TOCSY NMR spectra of compound **1,** three spin systems were observed at δ 4.10/44.5 (H1/C1) and δ 3.72/46.7 (H2/C2) (aliphatic spin system I), at δ 2.79/36.2 (H7″/C7″) and δ 4.88/73.5 (H8″/C8″) (aliphatic spin system II) and at δ 2.91/36.4 (H7A‴/C7‴), δ 2.87/36.4 (H7B‴/C7‴) and δ 4.98/73.3 (H8‴/C8‴) (Table 1). In the 2D ^1^H-^13^C HMBC spectrum of compound **1**, four ester carbonyl carbons were identified at δ 171.0 (C11), δ 165.4 (C12), δ 169.3 (C9″) and at δ 169.8 (C9‴). The carboxyl carbons C9″ and C9‴ were identified as methyl ester groups by observation of two 3H signals at δ 3.48 (9″-OCH_3_) and δ 3.52 (9‴-OCH_3_) observed in the 1D ^1^H NMR spectrum of 1, which exhibited crosspeaks at δ 3.48/169.3 (9″-OCH_3_/C9″) and δ 3.52/169.8 (9‴-OCH_3_/C9‴) observed in the 2D ^1^H-^13^C HMBC spectrum of compound **1**. The crosspeaks at δ 4.88/171.0 (H8″/C11) and δ 4.98/165.4 (H8‴/C12) observed in the 2D ^1^H-^13^C HMBC spectrum of compound **1** confirmed the linkages between the condensed caffeoyl units from which the molecular structure of compound **1** is comprised. The aromatic protons at δ 6.86 (s, H5) and δ 6.39 (s, H8) were assigned to a 2,3,5,6-tetrasubstituted aromatic moiety and the alkene proton at δ 7.56 (s, H4) was assigned as the β-proton of this substituted caffeoyl unit. The NMR data confirmed that compound **1** consists of a 1,2-dihydro-6,7-dihydroxy-1-(3,4-dihydroxyphenyl) naphthalene-2,3-dicarboxylic acid skeleton and two molecules of 3-(3,4-dihydroxyphenyl) lactic acid methyl ester (Table 1 and Figure 2) [8,9]. By using a combination of 1D ^1^H NMR in addition to 2D ^1^H-^13^C HMBC, 2D ^1^H-^13^C Edited HSQC, 2D ^1^H-^13^C HSQC-TOCSY, 2D ^1^H-^13^C H2BC, 2D ^1^H-^1^H COSY and 2D ^1^H-^1^H ROESY NMR experiments, all ^1^H and ^13^C signals of compound **1** were completely assigned (Table 1). Thus, compound **1** was identified as bis(3-(3,4-dihydroxyphenyl)-1-methoxy-1-oxopropan-2-yl)-1-(3,4-dihydroxyphenyl)-6,7-dihydroxy-1,2-dihydronaphthalene-2,3-dicarboxylate (Table 1 and Figure 2). A molecular ion [MH^+^] at m/z 747.18455 corresponding to C_38_H_35_O_15_ (calculated: 747.19167; δ = −9.54 ppm) observed in the high-resolution mass spectrum of compound **1** confirmed this identification (Figure S15). The fragment ion at *m*/*z* 535.11839, which corresponds to the loss of a dihydrocaffeoyl methyl ester moiety from the molecular ion, and the fragment ions at *m*/*z* 295.0558 and *m*/*z* 195.05574, which correspond to the loss of both dihydrocaffeoyl methyl ester moieties from the molecular ion, and an ionized dihydrocaffeoyl methyl ester moiety, respectively, (Figure S15) further supported the structure determination of compound **1**. The UV and NMR data of compound **1** are similar to those reported by Agata et al. for the analogous compound rabdosiin [8,9]. The negative band at 250 nm observed in the CD spectrum of **1** is analogous to that of the structurally closely related compound (+)-Rabdosiin reported by Fedoreyev et al. (2005) [10], indicating that these compounds have similar stereochemistry. Rabdosiin has also been identified in the related plant species *Echium russicum* [11] and *Echium plantagineum* [12]. Compound **1**, which is synonymous to rabdosiin dimethyl ester, has previously only been reported to be isolated from leaves of *Mentha haplocalyx* in a patent by Matano et al. (1993) [13]. To our knowledge, the content of this patent has never been published in any scientific journal. However, NMR data have not previously been published for this compound. Matano et al. (1993) reported that compound **1** and structurally related compounds exhibited significant anti-inflammatory activity [13]. The compounds were tested as inhibitors against 3α-hydroxysteroid dehydrogenase, which is known to be inhibited by anti-inflammatory drugs [14,15]. Compound **1** showed IC_50_ of 45.1 µg/mL against 3α-hydroxysteroid dehydrogenase which is significantly more potent than the known anti-inflammatory drug aspirin, with an IC_50_ value of 1150 µg/mL [13]. The crude extract of *E. amoenum* is comprised of a relatively complex mixture of polyphenolic compounds. A total of 1 mg pure compound **1** was obtained from 800 g fresh leaves of *E. amoenum*, indicating that the compound is not the main polyphenolic constituent of leaves from *E. amoenum*. However, the discovery of this compound in leaves of *E. amoenum* may be important in order to rationalize the traditional use of the plant in the treatment of inflammations.

The UV spectrum of compound **2** recorded on-line during HPLC analysis exhibited λ_max_ values at 242 nm, 300 nm (sh) and 326 nm, which is in accordance with an aromatic compound with a cinnamoyl type chromophore. The aromatic region of the 1D ^1^H NMR spectrum of compound **2** (Figure S3) showed a 3H ABX system at δ 7.04 (d 2.0 Hz, H2), δ 6.99 (dd 8.2, 2.0 Hz, H6) and δ 6.75 (d 8.2 Hz, H5), a 2H AX system at δ 7.48 (d 15.9 Hz, H7) and δ 6.24 (d 15.9 Hz, H8), as well as to two phenolic hydroxyl protons at δ 9.59 (4-OH) and δ 9.13 (3-OH), in accordance with a caffeoyl moiety. This identification was confirmed by the observation of nine ^13^C signals assigned to this unit by the 2D ^1^H-^13^C HMBC spectrum and the 2D ^1^H-^13^C HSQC spectrum of compound **2** (Table 1, Figures S4 and S5). The large coupling constant of 15.9 Hz observed for H7 and H8 confirmed the (*E*)-configuration of the double bond of the caffeoyl moiety. The crosspeaks at δ 4.11/166.4 (H1A’/C9) and δ 4.08/166.4 (H1B’/C9) revealed that the carboxyl of compound **2** was esterified with the γ-hydroxyl of a highly hydroxylated organic acid containing four carbons, which was identified as the methyl ester of threonic acid. A comparison between the relatively weak CD spectrum of compound **2** to that of commercially available L-threonic acid provided by Sigma Aldrich (Figure S16) revealed that both molecules exhibited a positive band around 195 nm and a negative band around 220 nm, indicating that the methyl threonyl moiety of compound **2** shares the same stereochemistry as that of L-threonic acid, which is synonymous with (2R,3S)-2,3,4-trihydroxybutanoic acid. Thus, compound **2** was identified as 4-Oxy-(*E*)-Caffeoyl-2′R,3′S-2,3-dihydroxybutanoic acid methyl ester (Figure 2). Moreover, the sample of compound **2** also contained a minor component, namely compund **3**. The ^1^H and ^13^C signals belonging to compound **3** shared many similarities with that of compound **2**, indicating that compound **3** is a structural isomer of compound **2**, i.e., a caffeoyl derivative esterified with threonic acid (Table 1). The coupling constant observed between H7 and H8 (12.9 Hz) confirmed the identity of compound **3** to be 4-Oxy-(*Z*)-Caffeoyl-2,3-dihydroxybutanoic acid methyl ester (Figure 2). Very recently, Cao et al. identified 4-Oxy-(*E*)-Caffeoyl-2′R,3′R-2,3-dihydroxybutanoic acid methyl ester as a novel natural product from the flower buds of *Magnolia biondii* pamp [16]. The UV and the ^1^H and ^13^C NMR data of compound **2** are in good agreement with the NMR data reported for the similar compound by Cao et al. (2021), with only relatively minor differences of chemical shift values due to solvent differences because the NMR data of Cao et al. were recorded in a different solvent (CD_3_OD) [16].

Compounds **4** and **5** were identified as (*Z*)-caffeic acid and rosmarinic acid methyl ester by a combination of 1D and 2D NMR spectroscopic techniques (Figure 2 and Supplementary Materials). These compounds have previously been identified in flowers of *E. amoenum* [2,17,18].

According to Nouri et al. (2019) [19], the effectiveness of *E. amoenum* in the treatment of anxiety, depression, ischemic stroke, seizure, Alzheimer’s disease and pain has been studied. Only the antidepressant and anxiolytic properties of the plant extract have been studied both clinically and experimentally [19]. Many of these effects have been attributed to the content of polyphenolic compounds such as rosmarinic acid, as well as anthocyanins and other flavonoids. Anthocyanins are exclusively present in the flowers of *E. amoenum*. The main anthocyanins have been identified as the relatively common compounds cyanidin 3-glucoside and delphinidin 3-glucoside [19]. Even though specific polyphenolic compounds identified in extracts of *E. amoenum* have been considered to be responsible for the observed biological activities, the studies performed so far have only been performed on plant extracts and not on pure compounds isolated from *E. amoenum*. It may also be mentioned that the polyphenolic compounds previously isolated from *E. amoenum* are relatively common natural products, which are present in several plant species and are not specific to *E. amoenum*. There is a strong motivation to isolate, identify and utilize the pure active constituents of *E. amoenum* because of the toxicity of, particularly, extracts of flowers of *E. amoenum*, which is attributed to the content of toxic pyrrolizidine alkaloids [20]. In this paper, we have demonstrated that *E. amoenum* indeed contains natural products with very restricted occurrence in nature.

## 3. Materials and methods

### 3.1. Plant Material

Seeds of *E. amoenum* were bought at the market in Teheran and cultivated in the Bergen Botanical Garden of the University of Bergen, Norway (coordinates 60.2499995 N 005.5191002 E). Fresh plant material was collected in August 2014 at the botanical garden. The leaves were separated and stored at −20 °C prior to extraction. 

### 3.2. Extraction of Compounds and Partitions with Organic Solvents 

Fresh leaves of *E. amoenum* (800 g) were extracted with 3.5 L methanol (MeOH) for 72 h at room temperature. The concentrated extract (440 mL) was purified to partition (twice) against equal volumes of petroleum ether and ethyl acetate, respectively. 

### 3.3. Amberlite XAD-7 Column Chromatography

The residual water phase of the extract was further purified on an Amberlite XAD-7 column. The mobile phase consisted of 5 L distilled water, followed by 1 L MeOH-H_2_O 10:90; *v*/*v*, 1 L MeOH-H_2_O 25:75; *v*/*v*, 1.5 L MeOH-H_2_O 50:50; *v*/*v*, 1 L MeOH-H_2_O 75:25; *v*/*v* and 3 L MeOH. Altogether, 20 fractions were collected. The fractions were analyzed by analytical HPLC. The combined fractions 7–10 were further separated by Sephadex LH-20 column chromatography.

### 3.4. Sephadex LH-20 Column Chromatography

Sephadex LH-20 column chromatography was performed on a 100 × 5 cm column. The mobile phase consisted of 2.1 L MeOH-H_2_O-trifluoroacetic acid (TFA) (20:80:0.2; *v*/*v*), followed by 1 L MeOH-H_2_O-TFA (40:60:0.2; *v*/*v*), 1 L MeOH-H_2_O-TFA (55:45:0.2; *v*/*v*), 1 L MeOH-H_2_O-TFA (65:35:0.2; *v*/*v*), 2 L MeOH-H_2_O-TFA (70:30:0.2; *v*/*v*), 1.3 L MeOH-H_2_O-TFA (73:27:0.2; *v*/*v*) and MeOH-H_2_O-TFA (76:24:0.2; *v*/*v*). Altogether, 41 fractions were collected. Fractions 22, 23, 27 and 37 were further separated by preparative HPLC.

### 3.5. Preparative HPLC

Compounds in fractions 22, 23, 27 and 37 from the Sephadex LH-20 separation were isolated by preparative HPLC. The HPLC instrument was equipped with a 250 × 22 mm, C_18_ Altech column. Two solvents were used for elution: A (water-TFA 99.5:0.5; *v*/*v*) and B (MeOH-TFA 99.5:0.5; *v*/*v*). The elution profile of the applied HPLC gradient is shown in Figure S2. The elution profile consisted of isocratic elution with MeOH-H_2_O-TFA (10:90:0.5 *v*/*v*/*v*) for 5 min, followed by a linear gradient from MeOH-H_2_O-TFA (10:90:0.5 *v*/*v*/*v*) to MeOH-H_2_O-TFA (40:60:0.5 *v*/*v*/*v*) for the next 40 min, isocratic elution with MeOH-H_2_O-TFA (40:60:0.5 *v*/*v*/*v*) for the next 15 min, followed by linear gradient from MeOH-H_2_O-TFA (40:60:0.5 *v*/*v*/*v*) to MeOH-H_2_O-TFA (70:30:0.5 *v*/*v*/*v*) for the next 10 min, followed by isocratic elution with MeOH-H_2_O-TFA (70:30:0.5 *v*/*v*/*v*) for the next 18 min. The flow rate was 14 mL m^−1^. The samples were dissolved in a total of 0.2–1.4 mL solvent. Aliquots of 200 μL of each of the samples were manually injected into the HPLC column. Each peak in the chromatogram was separately collected in vials. A total of 1–1.5 mL of each of the collected fractions was transferred to HPLC vials for later identifications using analytical HPLC. Following this strategy, 1 mg of compound **1**, 1.3 mg of compound **2**, 1.4 mg of compound **4** and 6 mg of compound **5** were isolated.

### 3.6. Analytical HPLC 

The HPLC instrument was equipped with a multidiode array detector, an autoinjector and a 250 × 4.6 mm, 5 μm Thermo Scientific Hypersil GOLD column. Two solvents were used for elution: A (water-TFA 99.5:0.5; *v*/*v*) and B (acetonitrile-TFA 99.5:0.5; *v*/*v*). The elution profile of the applied HPLC gradient is shown in Figure S1. The elution profile consisted of initial conditions with 90% A and 10% B, followed by gradient elution for 10 min (14%B), isocratic elution for 10–14 min, and the subsequent gradient conditions: 18 min (16% B), 22 min (18% B), 26 min (23% B), 31 min (28% B), and 32 min (40% B), isocratic elution 32–40 min, gradient elution 40–43 min (10% B), and final isocratic elution 43–46 min (10% B). The analytical HPLC pump system was purged with both solution A (super distilled water and 0.5% TFA) and solution B (acetonitrile and 0.5% TFA) for 15 min each with a flow of 5 mL/min. The column was thereafter equilibrated with a flow of 1 mL/min in 30 min with acetonitrile-super distilled water (10:90 *v*/*v*). A total of 20 μL of each sample was injected with an autoinjector. The flow rate was 1 mL/min. 

### 3.7. Spectroscopy 

High resolution mass spectra were recorded using a JEOL AccuTOF^TM^ mass spectrometer operated in positive mode at a resolving power of approximately 6000 FWHM. The atmospheric pressure interface zone was tuned for the optimization of ions below *m*/*z* 1000 and an electric potential of 2500 V (needle voltage) was applied. The TOF mass selection window was set to detect *m*/*z* values up to 2000, and the mass spectral acquisition settings applied were as follows: spectral recording interval = 0.5 s, wait time = 0.03 ns and data sampling interval = 0.5 ns. The samples were analysed as solutions in acetonitrile (~50 µg/mL) and introduced to the ESI spray chamber by weakly acidified (0.01% HCOOH) acetonitrile used as spray reagent. Internal mass drift calibration was performed using a 1 ppm solution of PEG600 (polyethylene glycol average mass 600 u) in acetonitrile. 

UV-Vis absorption spectra were recorded on-line during HPLC analysis over the wavelength range 240–600 nm in steps of 2 nm.

CD spectra from 180 to 260 nm (light path 1 mm) of the compounds in MeOH were recorded at 20 °C in a Jasco J-810 spectropolarimeter equipped with a Peltier temperature control unit. The spectra obtained were the average of four scans, and MeOH buffer scans were subtracted from the obtained spectra. 

NMR samples were prepared by dissolving the isolated compounds in deuterated dimethylsulfoxide (DMSO-D_6_; 99.95 atom % D, Sigma-Aldrich, Saint Louis, MO, USA). The 1D ^1^H and the 2D ^1^H-^13^C HMBC, the 2D ^1^H-^13^C HSQC, the 2D ^1^H-^13^C HSQCTOCSY, the 2D ^1^H-^13^C H2BC, the 2D ^1^H-^1^H COSY and 2D ^1^H-^1^H ROESY NMR experiments were obtained at 600.13 MHz and 150.90 MHz for ^1^H and ^13^C, respectively, at 298K on a Bruker 600 MHz instrument equipped with a ^1^H,^13^C,^15^N triple resonance cryogenic probe.

## 4. Conclusions

For the first time, polyphenolic compounds of leaves of *E. amoenum* have been isolated and identified. The fact that compound **1** belongs to a rare group of natural products which have previously been patented for their significant anti-inflammatory activity by Matano et al. (1993) [16] may rationalize the traditional treatment of inflammations with *E. amoenum*. 

## Figures and Tables

**Table 1:** ^1^H and ^13^C NMR chemical shift values (ppm) and coupling constants (Hz) of compounds **1**–**3** in DMSO-D_6_ at 298 K.

	1 δ ^1^H	1 δ ^13^C	2 δ ^1^H	2 δ ^13^C	3 δ ^1^H	3 δ ^13^C
1	4.10 d 2.4	44.5		125.5		126.0
2	3.72 d 2.4	46.7	7.04 d 2.0	114.9	7.37 d 2.0	117.8
3		119.4		145.4		144.8
4	7.56 s	139.4		148.4		147.2
5	6.86 s	116.7	6.75 d 8.2	115.8	6.71 d 8.2	115.0
6		144.4	6.99 dd 2.0, 8.2	121.5	7.05 dd 2.0, 8.2	123.7
7		148.3	7.48 d 15.9	145.5	6.77 d 12.9	143.8
8	6.39 s	116.4	6.24 d 15.9	113.8	5.73 d 12.9	114.8
9		129.3		166.4		165.8
10		122.4				
11		171.0				
12		165.4				
1A’		134.3	4.11 m	64.5	4.08 m	n.a
1B’			4.08 m			
2′	6.26 d 2.3	114.7	4.02 m	69.7	3.98 m	69.7
3′		144.9	4.17 dd 2.7, 7.2	71.3	4.12 m	71.3
4′		144.1		172.9		172.9
4′-OCH_3_			3.64 s	51.6	3.64 s	51.6
5′	6.56 d 8.2	115.6				
6′	6.21 dd 2.3, 8.2	117.9				
1″		126.5				
2″	6.57 d 2.1	116.6				
3″		145.1				
4″		144.2				
5″	6.61 d 8.0	115.5				
6″	6.42 dd 2.1, 8.0	120.3				
7″	2.79 d 6.7	36.2				
8″	4.88 t 6.7	73.5				
9″		169.3				
9″-OCH_3_	3.48 s	52.0				
1‴		126.4				
2‴	6.60 d 2.1	116.8				
3‴		145.1				
4‴		144.2				
5‴	6.63 d 8.0	115.5				
6‴	6.43 dd 2.1, 8.0	120.3				
7a‴	2.91 dd 6.8, 14.1	36.4				
7b‴	2.87 dd 5.9, 14.1					
8‴	4.98 dd, 5.9, 6.8	73.3				
9‴		169.8				
9‴-OCH_3_	3.52 s	51.9				
3-OH			9.13 s		9.04 s	
4-OH			9.59 s		9.41 s	
6-OH	9.09 s					
7-OH	9.45 s					
2′-OH			5.18 d 7.0		5.16 m	
3′-OH	8.73 s		5.34 d 7.2		5.32 d 7.2	
4′-OH	8.69 s					
3″-OH	8.75 s					
4″-OH	8.726 s					
3‴-OH	8.80 s					
4‴-OH	8.74 s					

## Data Availability

Data is contained within the article or Supplementary Material.

## Supplementary Materials

The following supporting information can be downloaded at: https://www.mdpi.com/article/10.3390/molecules28010385/s1, Figure S1: Gradient of solvents; (A) super distilled water + 0.1% TFA and (B) acetonitrile + 0.1% TFA applied during isolation of compounds using analytical HPLC. Figure S2. Gradient of solvents; (A) super distilled water + 0.1% TFA and (B) MeOH + 0.1% TFA applied during isolation of compounds using preparative HPLC. Figure S3. 1D ^1^H NMR spectrum of 4-Oxy-(*E*)-caffeoyl-2,3-dihydroxybutanoic acid methyl ester (**2**). Figure S4. 2D ^1^H-^13^C HMBC NMR spectrum of 4-Oxy-(*E*)-caffeoyl-2,3-dihydroxybutanoic acid methyl ester (**2**). Figure S5. 2D ^1^H-^13^C Edited HSQC NMR spectrum of 4-Oxy-(*E*)-caffeoyl-2,3-dihydroxybutanoic acid methyl ester (**2**). Figure S6. 2D ^1^H-^1^H COSY NMR spectrum of 4-Oxy-(*E*)-caffeoyl-2,3-dihydroxybutanoic acid methyl ester (**2**). Figure S7. 2D ^1^H-^1^H ROESY NMR spectrum of 4-Oxy-(*E*)-caffeoyl-2,3-dihydroxybutanoic acid methyl ester (**2**). Figure S8. 1D ^1^H NMR spectrum of bis(3-(3,4-dihydroxyphenyl)-1-methoxy-1-oxopropan-2-yl)-1-(3,4-dihydroxyphenyl)-6,7-dihydroxy-1,2-dihydronaphthalene-2,3-dicarboxylate (**1**). Figure S9. 2D ^1^H-^13^C HMBC NMR spectrum of bis(3-(3,4-dihydroxyphenyl)-1-methoxy-1-oxopropan-2-yl)-1-(3,4-dihydroxyphenyl)-6,7-dihydroxy-1,2-dihydronaphthalene-2,3-dicarboxylate (**1**). Figure S10. 2D Edited ^1^H-^13^C HSQC NMR spectrum of bis(3-(3,4-dihydroxyphenyl)-1-methoxy-1-oxopropan-2-yl)-1-(3,4-dihydroxyphenyl)-6,7-dihydroxy-1,2-dihydronaphthalene-2,3-dicarboxylate (**1**). Figure S11. 2D ^1^H-^13^C HSQC-TOCSY NMR spectrum of bis(3-(3,4-dihydroxyphenyl)-1-methoxy-1-oxopropan-2-yl)-1-(3,4-dihydroxyphenyl)-6,7-dihydroxy-1,2-dihydronaphthalene-2,3-dicarboxylate (**1**). Figure S12. 2D ^1^H-^13^C H2BC NMR spectrum of bis(3-(3,4-dihydroxyphenyl)-1-methoxy-1-oxopropan-2-yl)-1-(3,4-dihydroxyphenyl)-6,7-dihydroxy-1,2-dihydronaphthalene-2,3-dicarboxylate (**1**). Figure S13. 2D ^1^H-^1^H COSY NMR spectrum of bis(3-(3,4-dihydroxyphenyl)-1-methoxy-1-oxopropan-2-yl)-1-(3,4-dihydroxyphenyl)-6,7-dihydroxy-1,2-dihydronaphthalene-2,3-dicarboxylate (**1**). Figure S14. 2D ^1^H-^1^H ROESY NMR spectrum of bis(3-(3,4-dihydroxyphenyl)-1-methoxy-1-oxopropan-2-yl)-1-(3,4-dihydroxyphenyl)-6,7-dihydroxy-1,2-dihydronaphthalene-2,3-dicarboxylate (**1**). Figure S15. High resolution mass spectrum of bis(3-(3,4-dihydroxyphenyl)-1-methoxy-1-oxopropan-2-yl)-1-(3,4-dihydroxyphenyl)-6,7-dihydroxy-1,2-dihydronaphthalene-2,3-dicarboxylate (**1**). Figure S16. Circular dichroism (CD) spectra of bis(3-(3,4-dihydroxyphenyl)-1-methoxy-1-oxopropan-2-yl)-1-(3,4-dihydroxyphenyl)-6,7-dihydroxy-1,2-dihydronaphthalene-2,3-dicarboxylate (**1**) and 4-Oxy-(*E*)-caffeoyl-2,3-dihydroxybutanoic acid methyl ester (**2**).
