# Supplementary material for: Natural Products from Leaves of the Ancient Iranian Medicinal Plant Echium amoenum Fisch. & C. A. Mey
Source: Molecules. 2023 Jan 2;28(1):385. doi: 10.3390/molecules28010385 (PMC9822446; doi:10.3390/molecules28010385)

Supplementary data

Natural products from leaves of the ancient Iranian medicinal plant *Echium amoenum*.

Mehdi Jafari Jamnani, Bjarte Holmelid, Anni Vedeler, Hoda H. Parsian,

Heidi Lie Andersen and Torgils Fossen

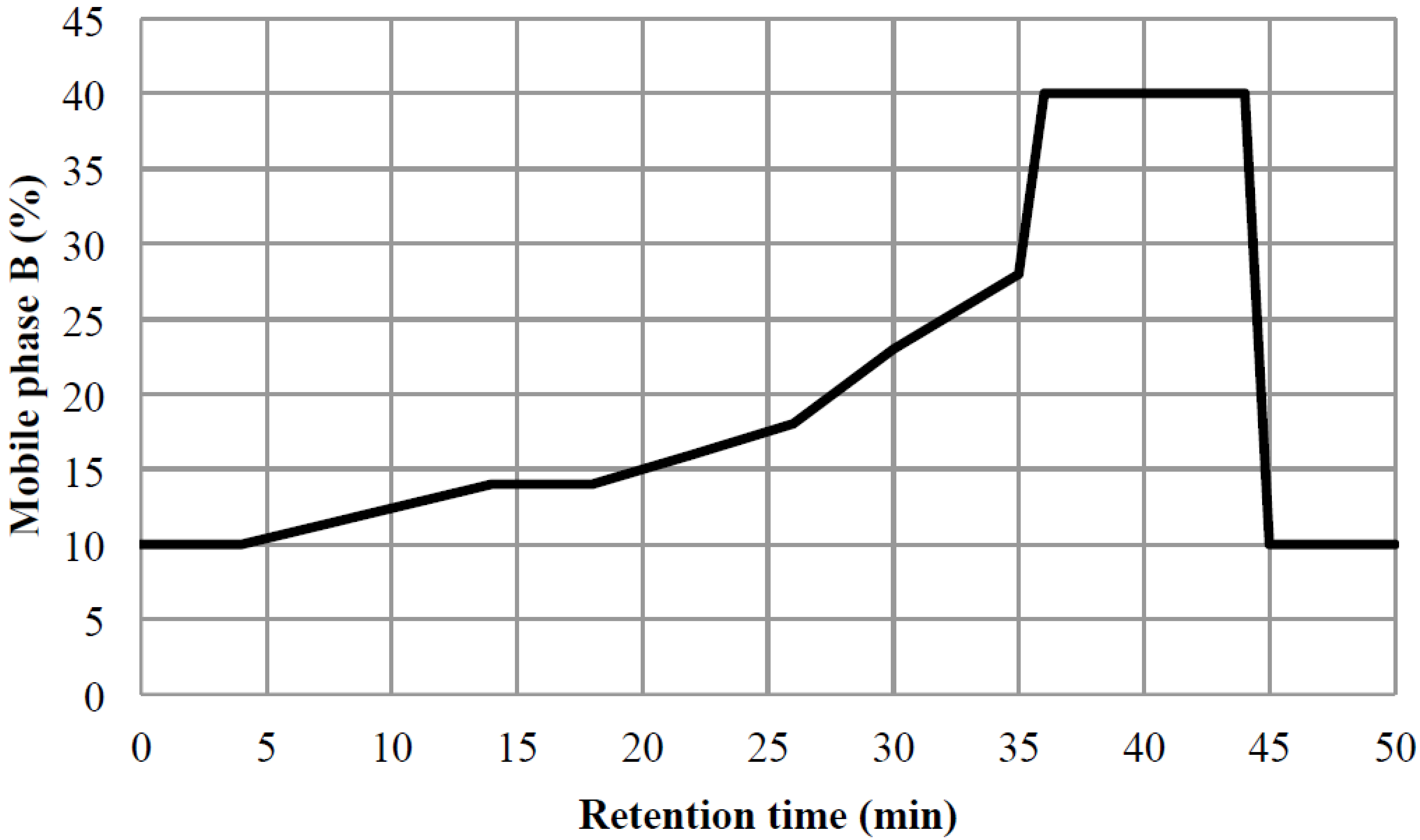

Figure S1. Gradient of solvents; (**A**) super distilled water + 0.1 % TFA and (**B**) acetonitrile + 0.1 % TFA) applied during isolation of compounds using analytical HPLC.

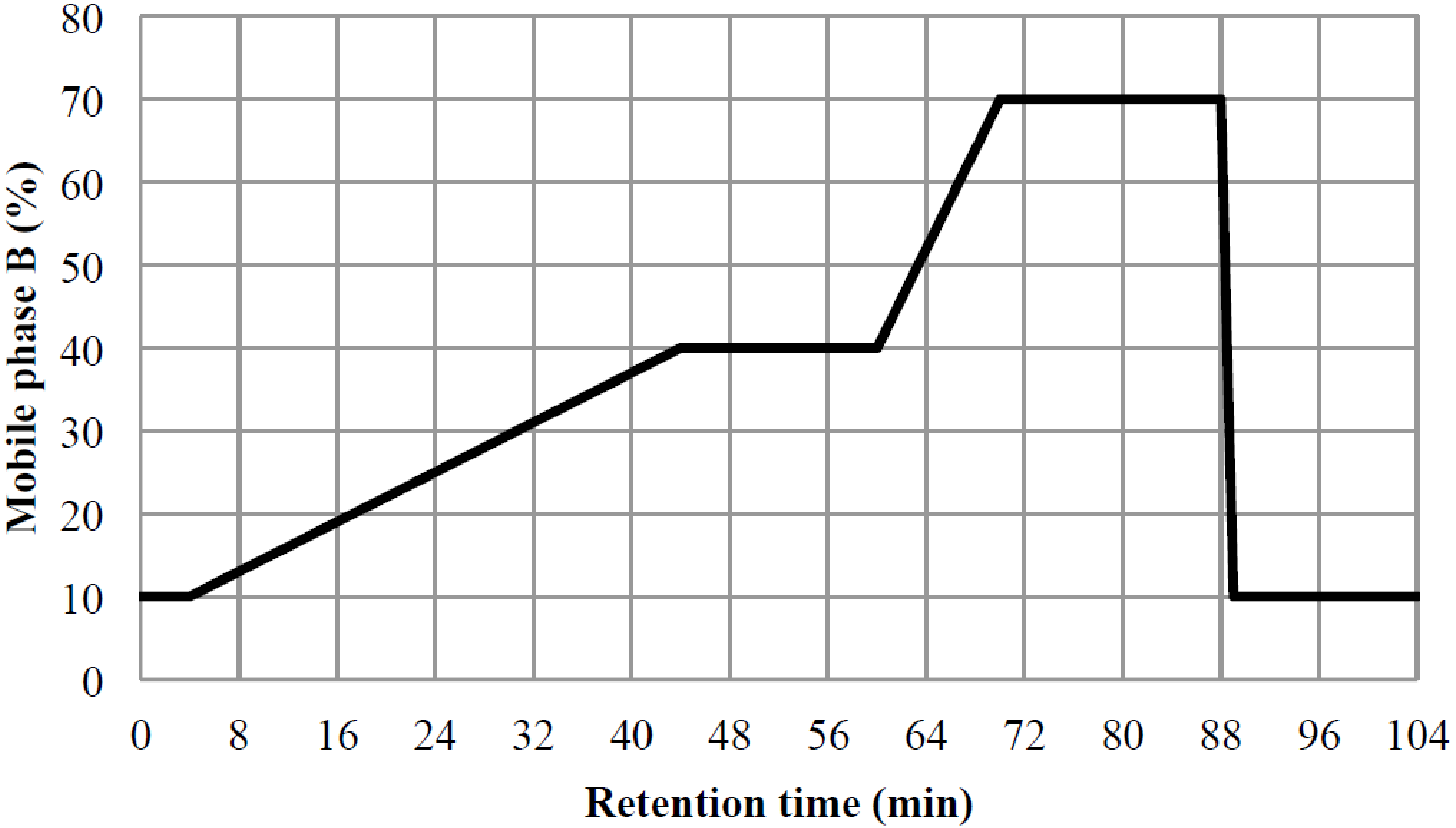

Figure S2. Gradient of solvents; (A) super distilled water + 0.1 % TFA and (B) methanol + 0.1 % TFA) applied during isolation of compounds using preparative HPLC.

Figure S3. 1D ^{1}H NMR spectrum of 4-Oxy-(E)-caffeoyl-2,3-dihydroxybutanoic acid methyl ester

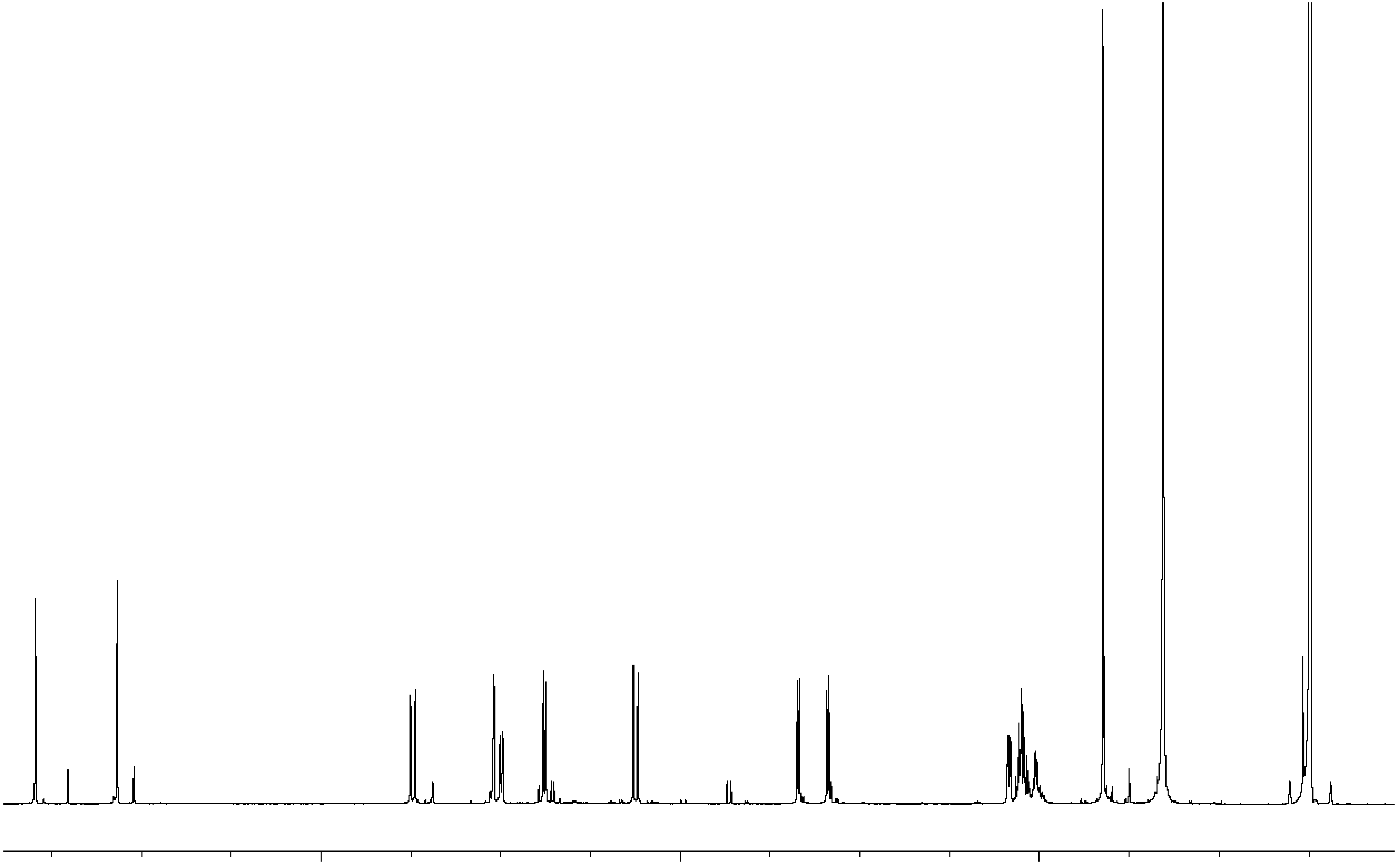

Figure S4. 2D ^{1}H-^{13}C HMBC NMR spectrum of 4-Oxy-(E)-caffeoyl-2,3-dihydroxybutanoic acid methyl ester

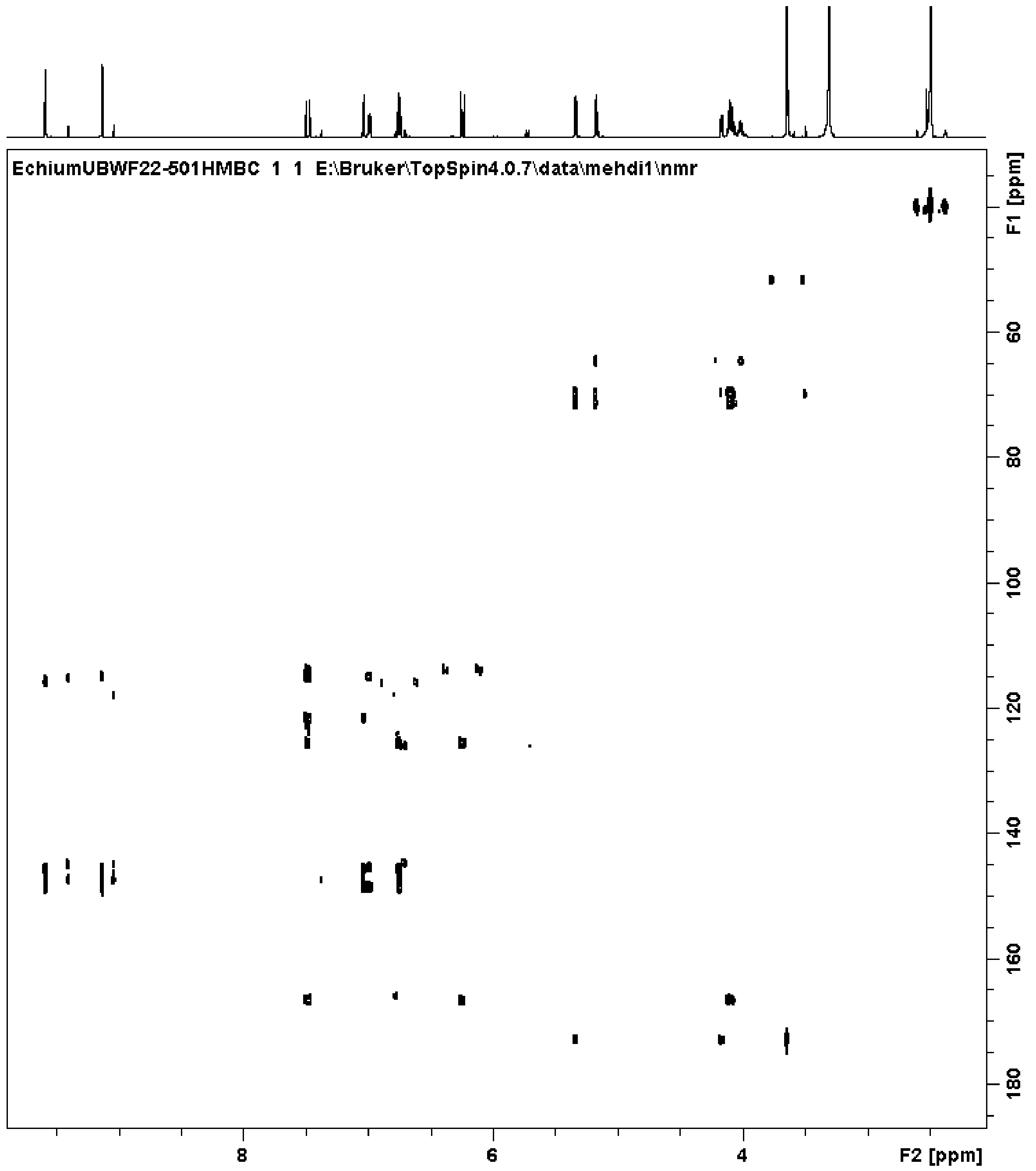

Figure S5. 2D ^{1}H-^{13}C Edited HSQC NMR spectrum of 4-Oxy-(E)-caffeoyl-2,3-dihydroxybutanoic acid methyl ester

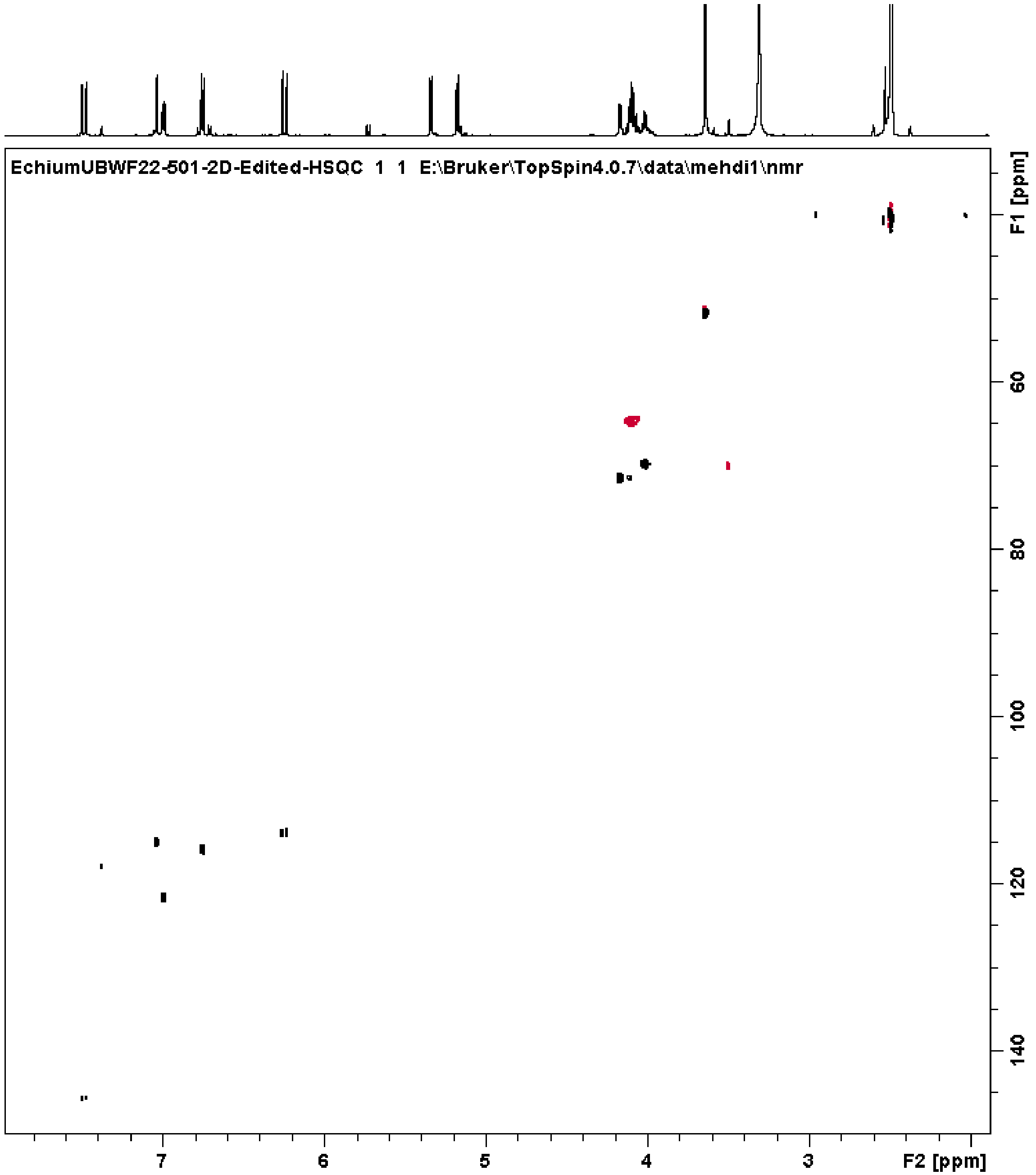

Figure S6. 2D ^{1}H-^{1}H COSY NMR spectrum of 4-Oxy-(E)-caffeoyl-2,3-dihydroxybutanoic acid methyl ester

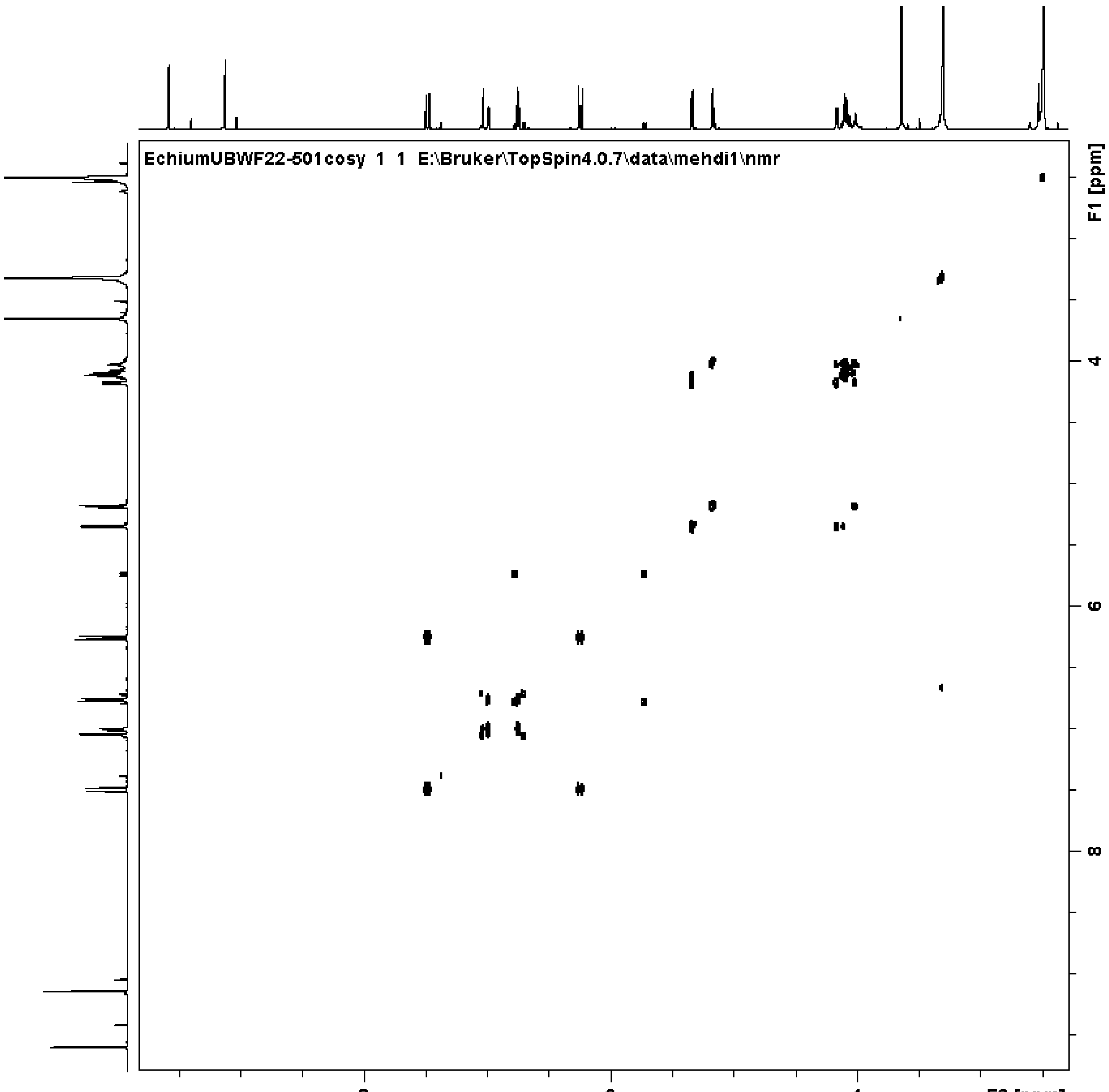

Figure S7. 2D ¹H-¹H ROESY NMR spectrum of 4-Oxy-(E)-caffeoyl-2,3-dihydroxybutanoic acid methyl ester

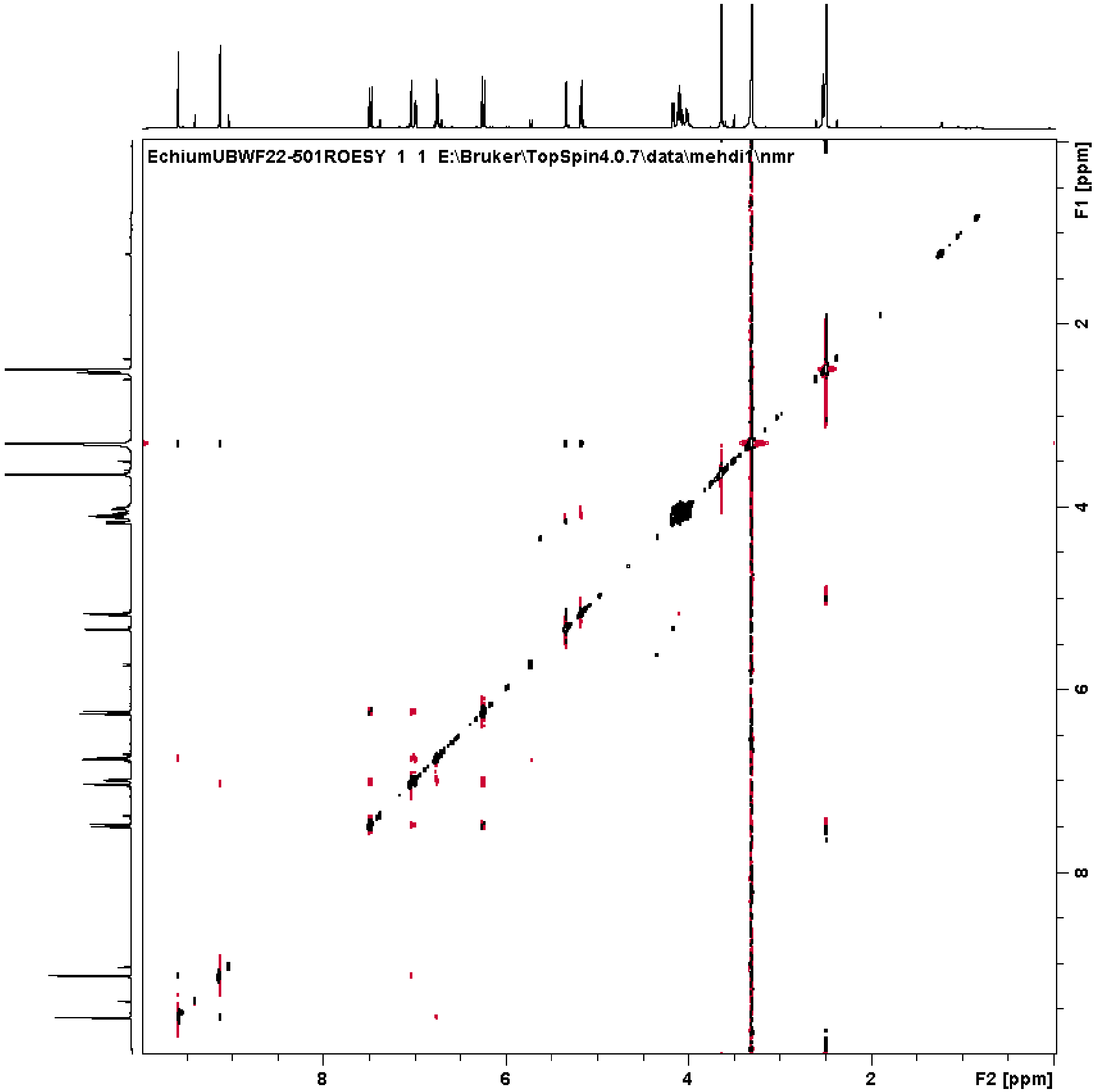

Figure S8. 1D ^1H NMR spectrum of bis(3-(3,4-dihydroxyphenyl)-1-methoxy-1-oxopropan-2-yl)-1-(3,4-dihydroxyphenyl)-6,7-dihydroxy-1,2-dihydronaphthalene-2,3-dicarboxylate

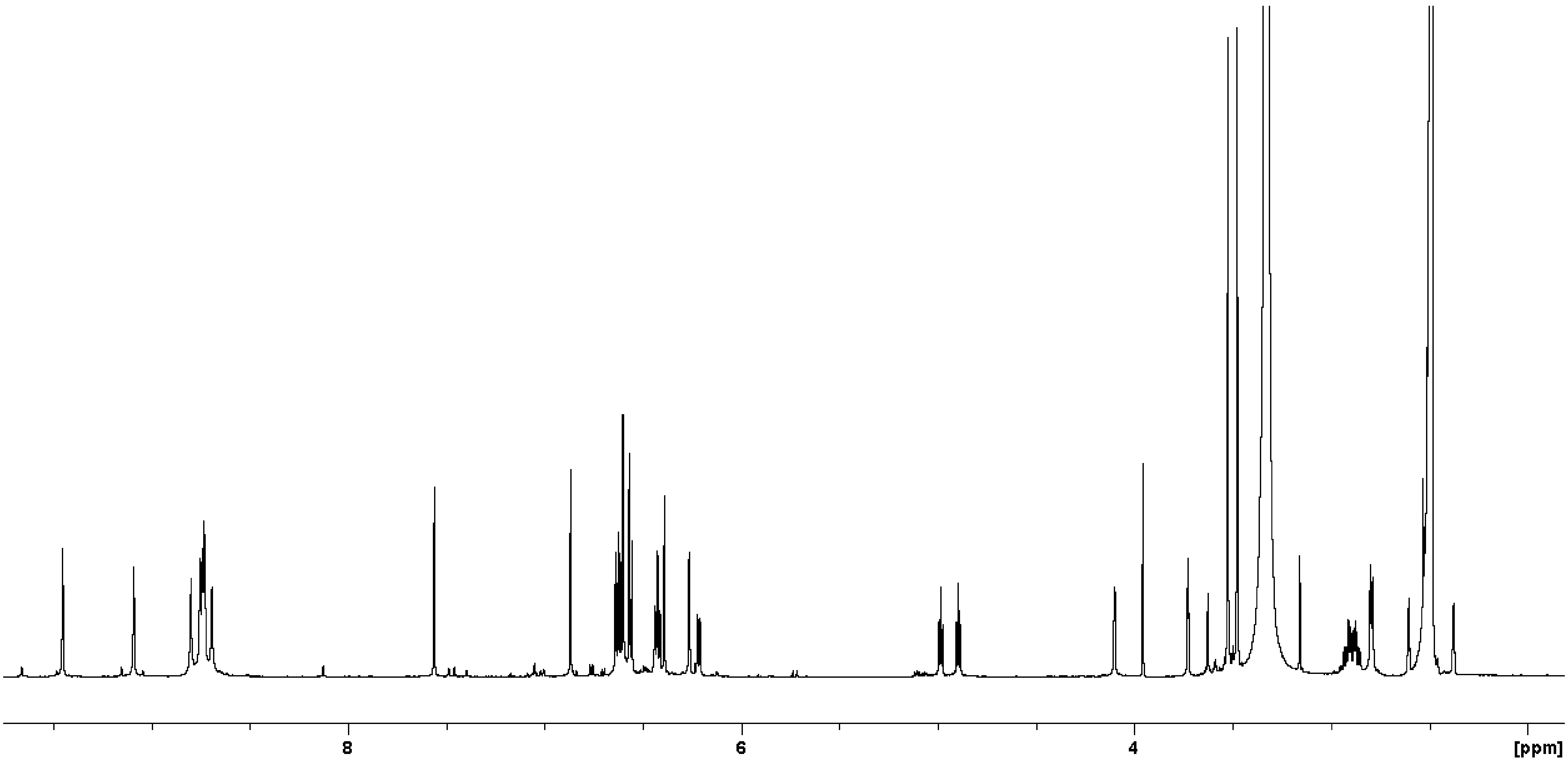

Figure S9. 2D ^{1}H-^{13}C HMBC NMR spectrum of bis(3-(3,4-dihydroxyphenyl)-1-methoxy-1-oxopropan-2-yl)-1-(3,4-dihydroxyphenyl)-6,7-dihydroxy-1,2-dihydronaphthalene-2,3-dicarboxylate

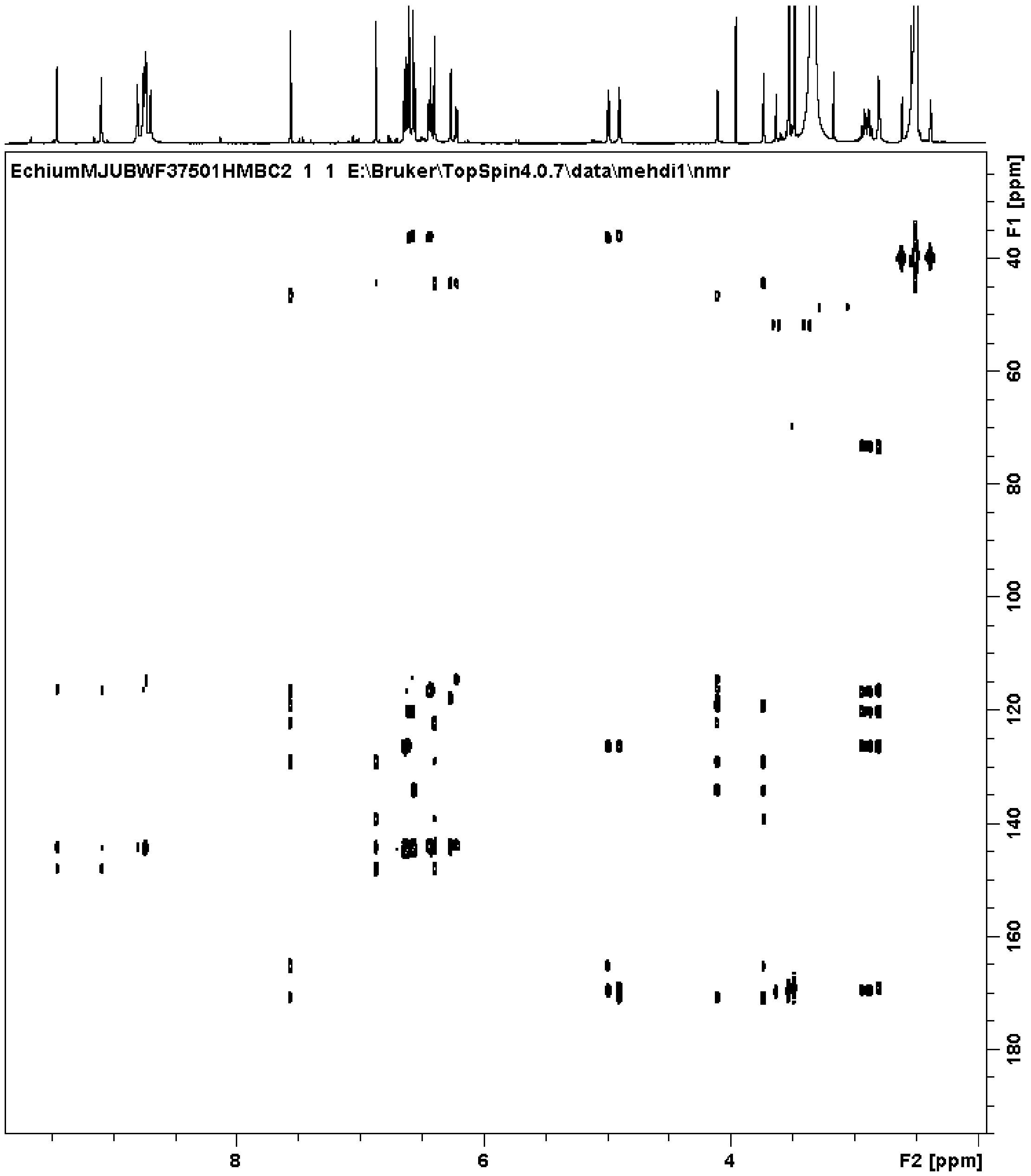

Figure S10. 2D Edited ^{1}H-^{13}C HSQC NMR spectrum of bis(3-(3,4-dihydroxyphenyl)-1-methoxy-1-oxopropan-2-yl)-1-(3,4-dihydroxyphenyl)-6,7-dihydroxy-1,2-dihydronaphthalene-2,3-dicarboxylate

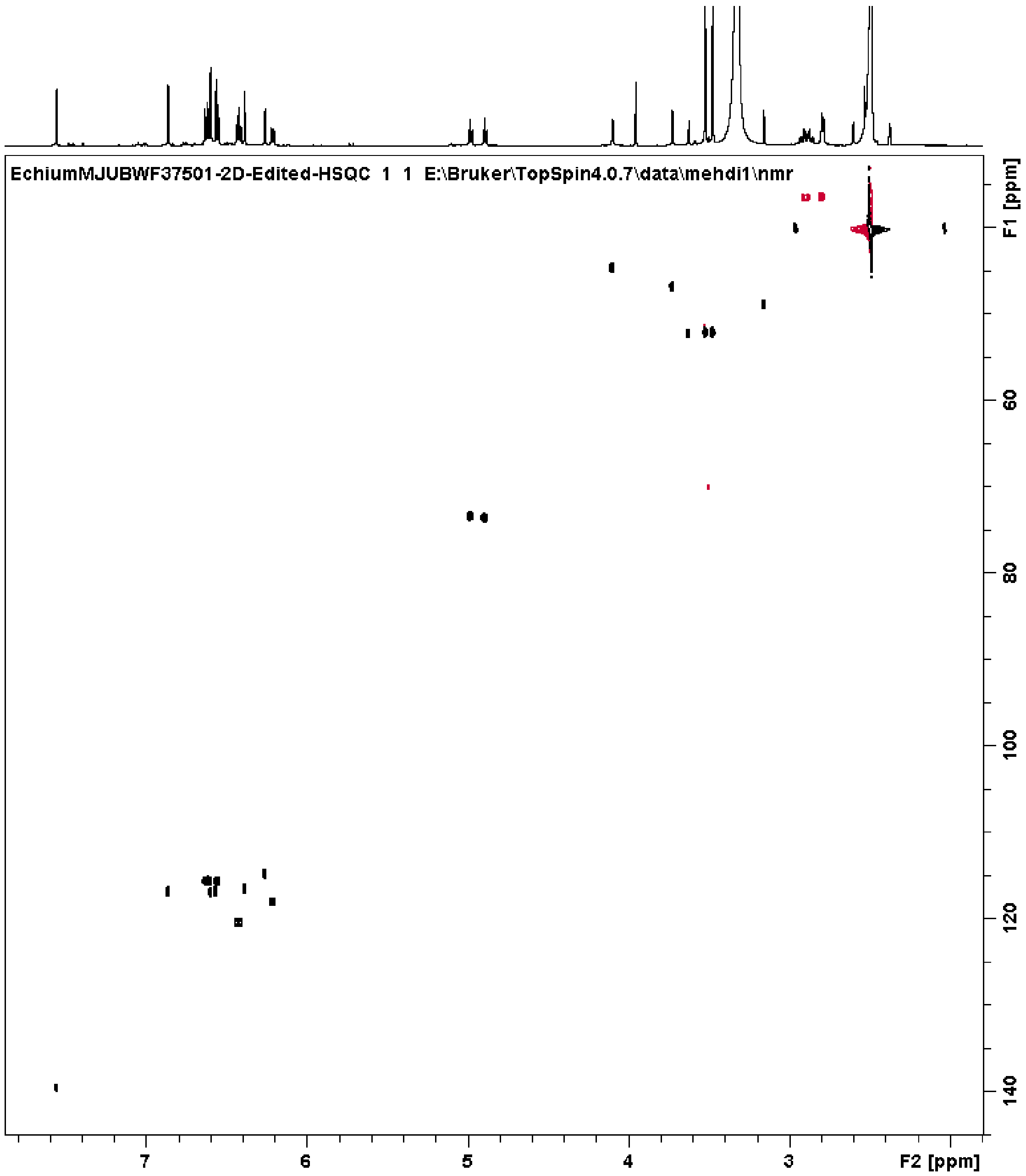

Figure S11. 2D ^{1}H-^{13}C HSQC-TOCSY NMR spectrum of bis(3-(3,4-dihydroxyphenyl)-1-methoxy-1-oxopropan-2-yl)-1-(3,4-dihydroxyphenyl)-6,7-dihydroxy-1,2-dihydronaphthalene-2,3-dicarboxylate

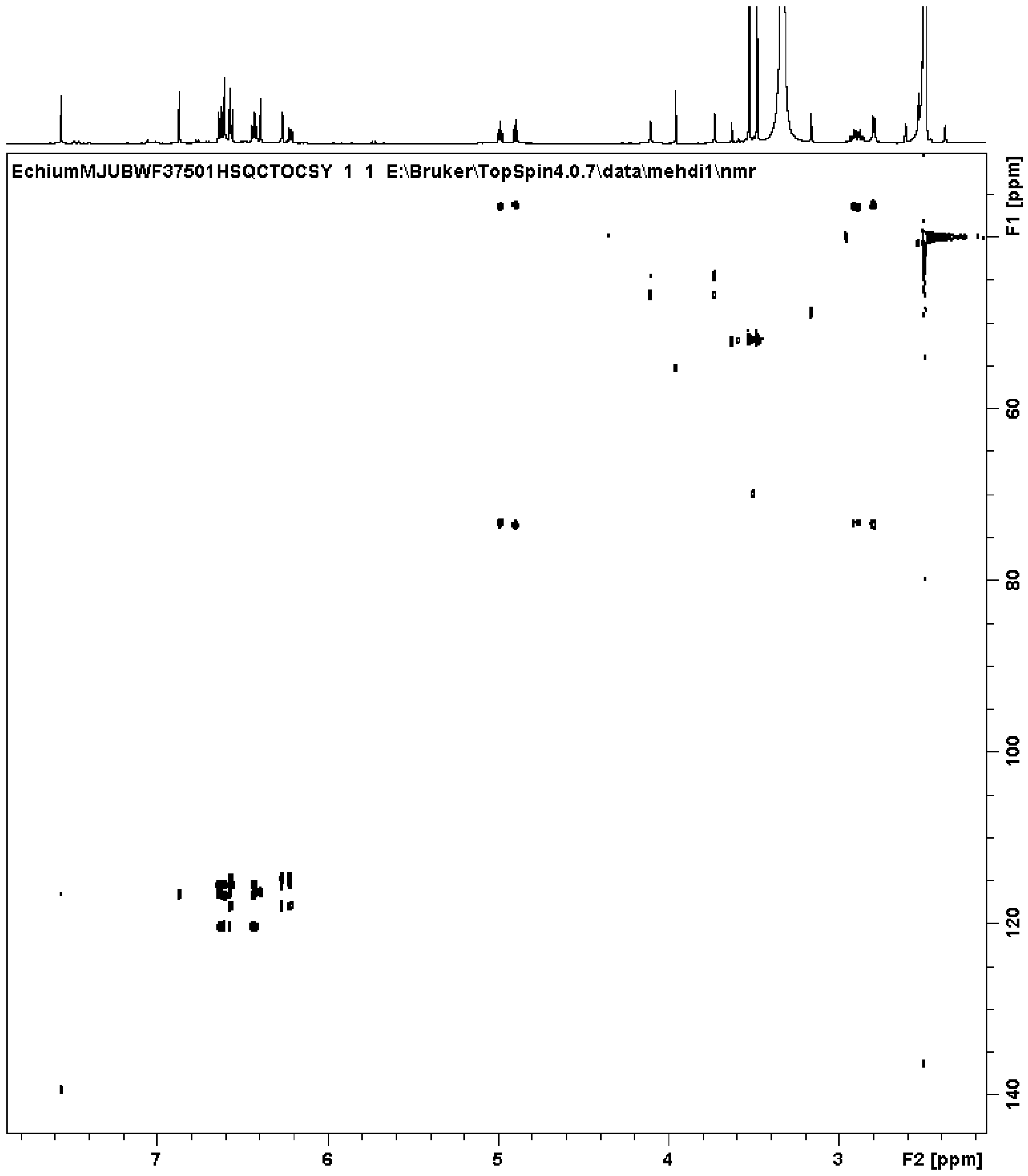

Figure S12. 2D ^{1}H-^{13}C H2BC NMR spectrum of bis(3-(3,4-dihydroxyphenyl)-1-methoxy-1-oxopropan-2-yl)-1-(3,4-dihydroxyphenyl)-6,7-dihydroxy-1,2-dihydronaphthalene-2,3-dicarboxylate

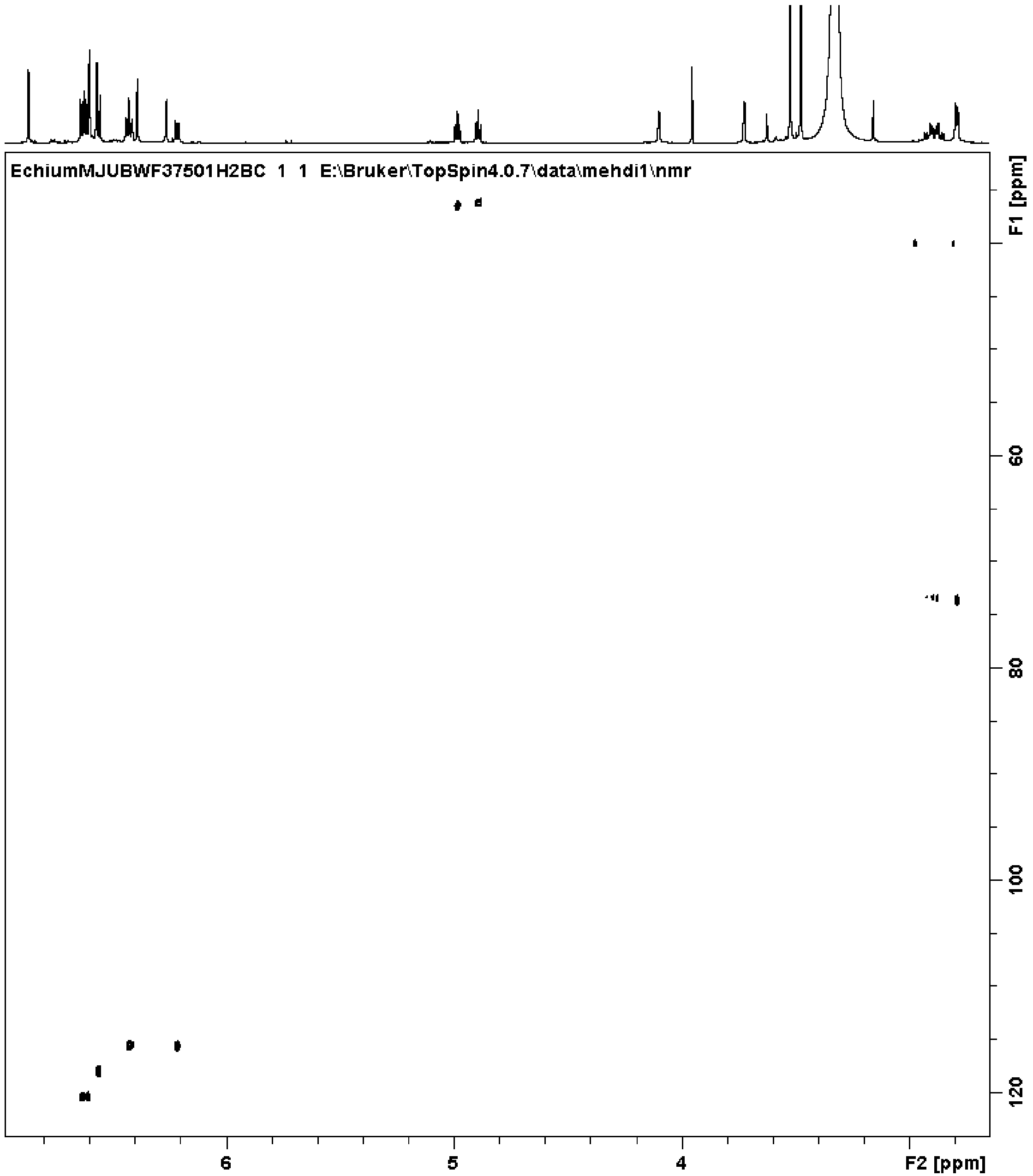

Figure S13. 2D ^{1}H-^{1}H COSY NMR spectrum of bis(3-(3,4-dihydroxyphenyl)-1-methoxy-1-oxopropan-2-yl)-1-(3,4-dihydroxyphenyl)-6,7-dihydroxy-1,2-dihydronaphthalene-2,3-dicarboxylate

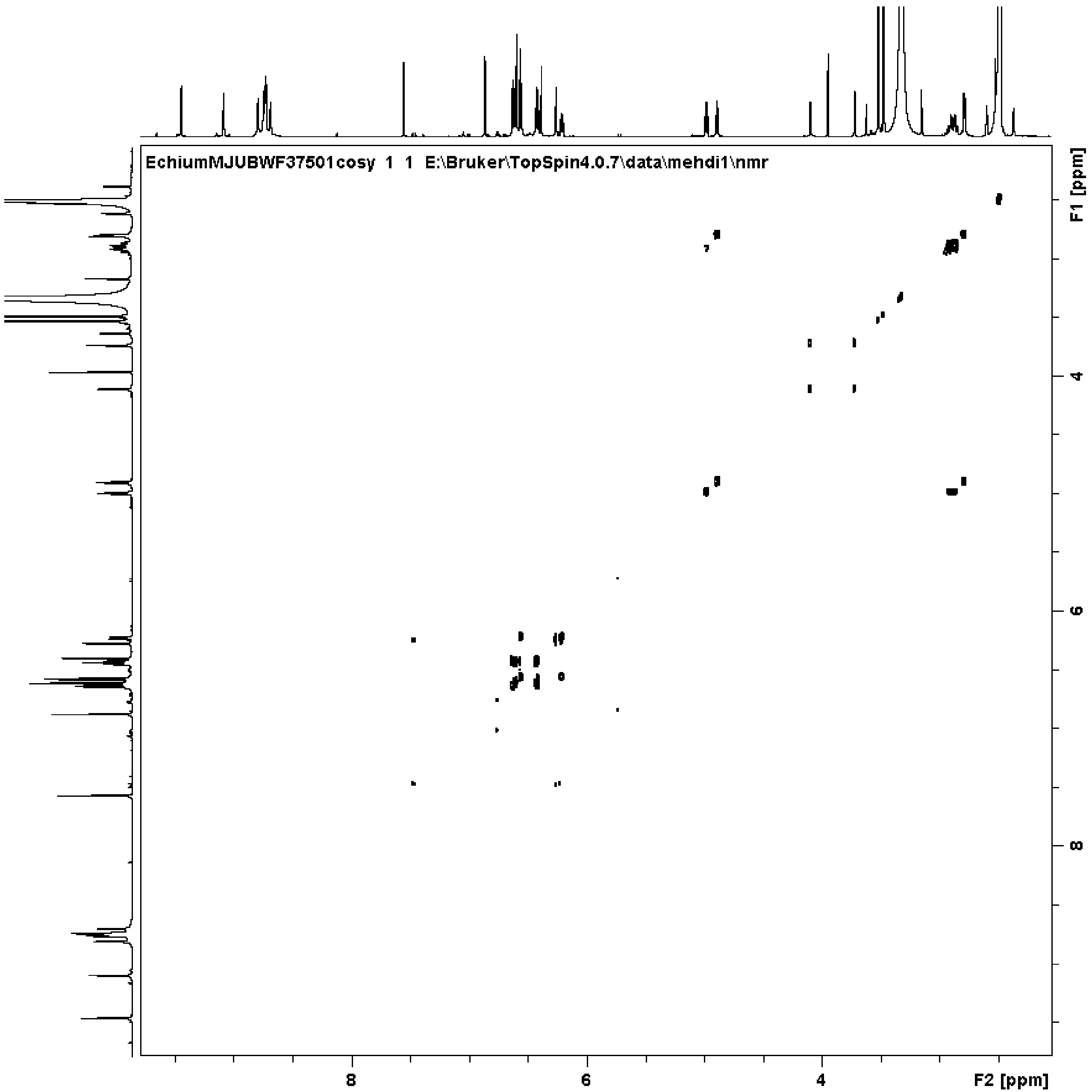

Figure S14. 2D ^{1}H-^{1}H ROESY NMR spectrum of bis(3-(3,4-dihydroxyphenyl)-1-methoxy-1-oxopropan-2-yl)-1-(3,4-dihydroxyphenyl)-6,7-dihydroxy-1,2-dihydronaphthalene-2,3-dicarboxylate

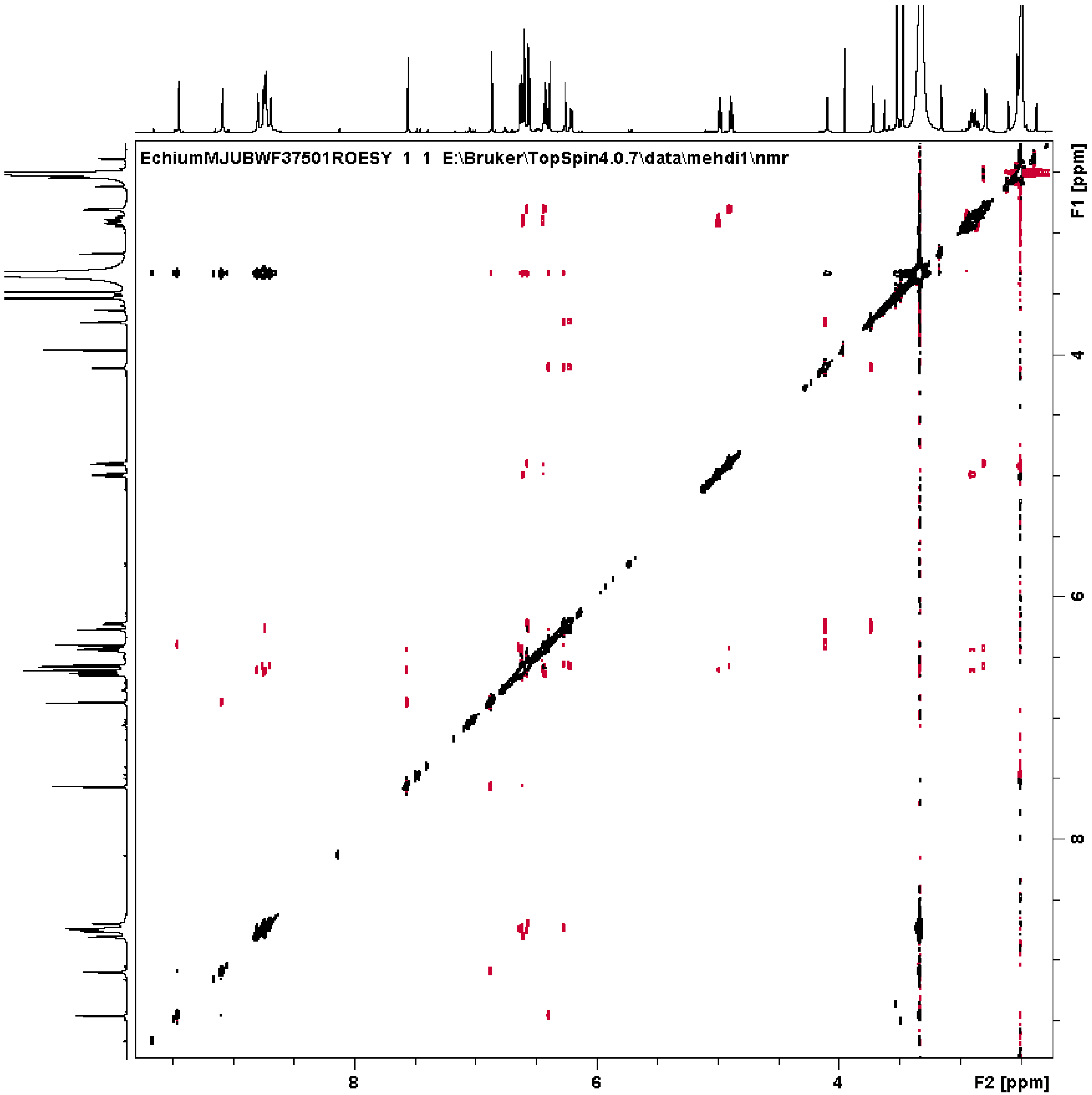

Figure S15. High resolution mass spectrum of bis(3-(3,4-dihydroxyphenyl)-1-methoxy-1-oxopropan-2-yl)-1-(3,4-dihydroxyphenyl)-6,7-dihydroxy-1,2-dihydronaphthalene-2,3-dicarboxylate

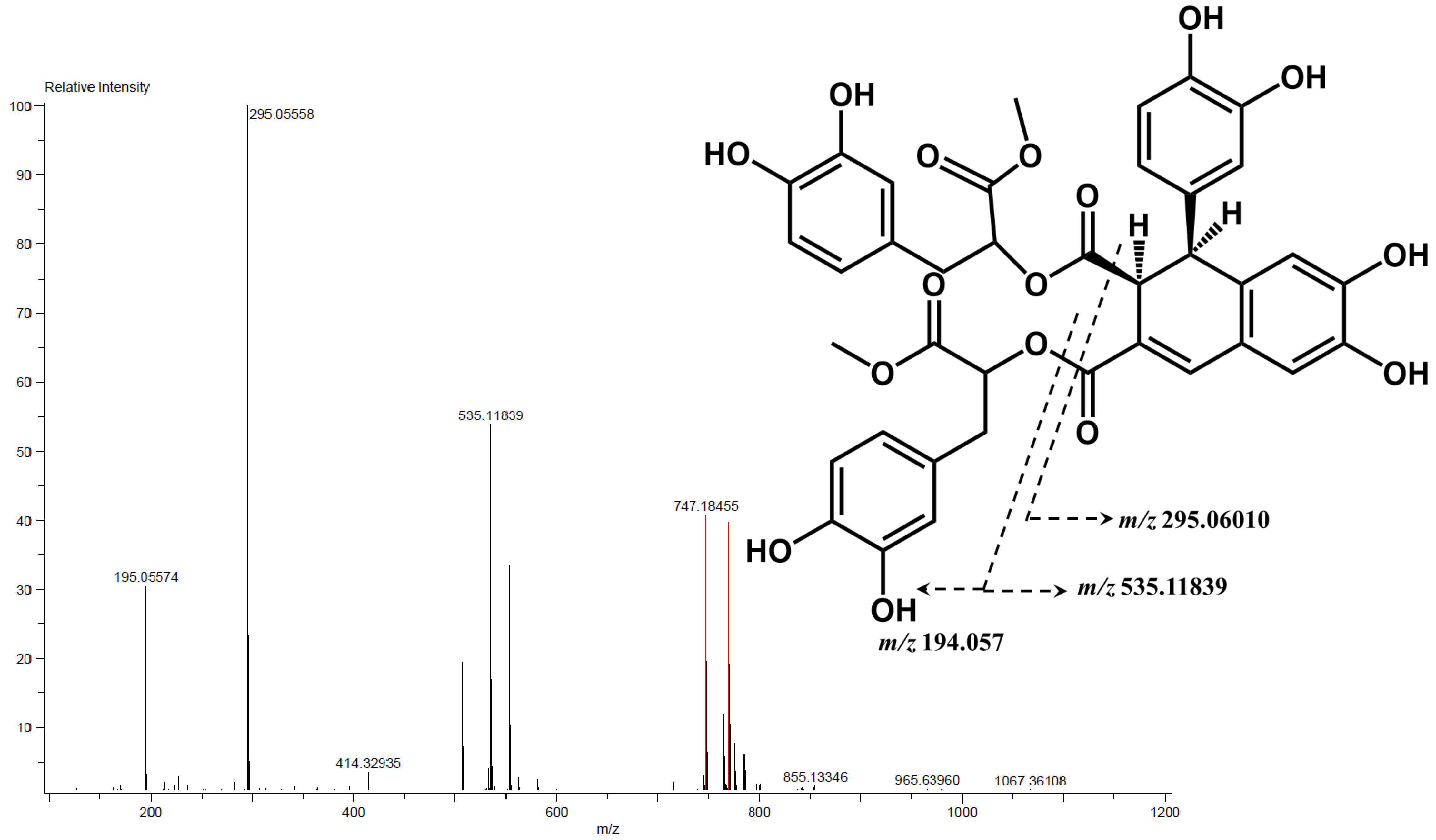

Figure S16. Circular dichroism (CD) spectra of bis(3-(3,4-dihydroxyphenyl)-1-methoxy-1-oxopropan-2-yl)-1-(3,4-dihydroxyphenyl)-6,7-dihydroxy-1,2-dihydronaphthalene-2,3-dicarboxylate (1) and 4-Oxy-(*E*)-caffeoyl-2,3-dihydroxybutanoic acid methyl ester (2)

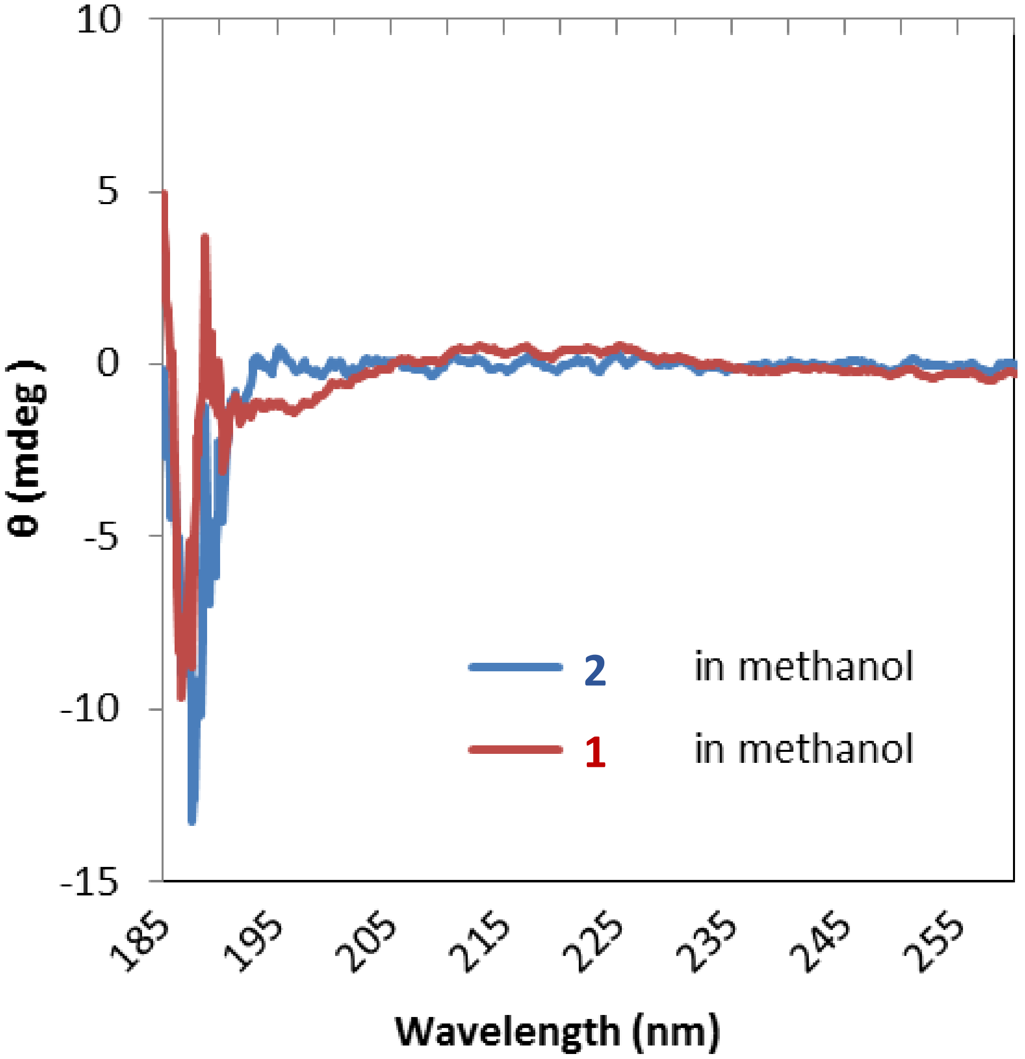

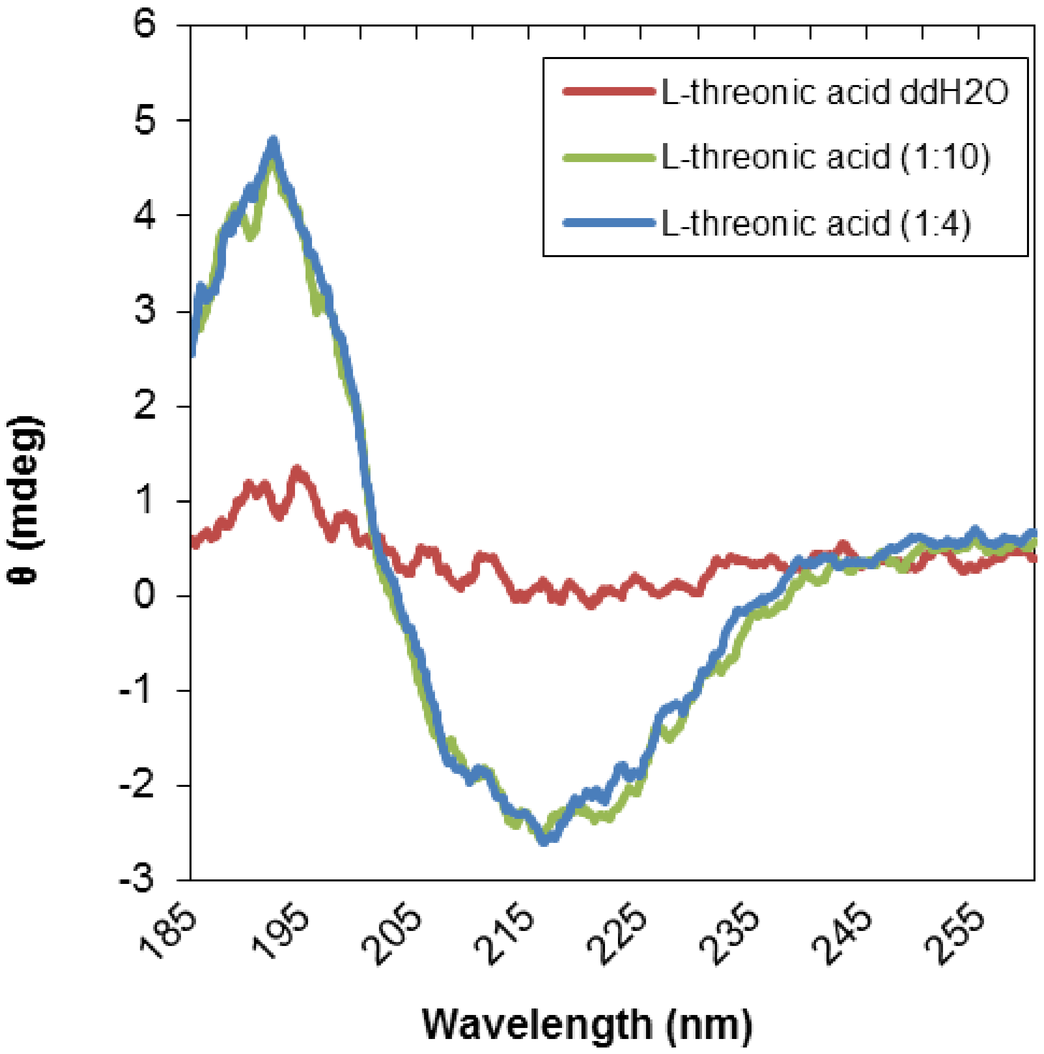

Figure S17. UV spectrum and HPLC chromatogram of bis(3-(3,4-dihydroxyphenyl)-1-methoxy-1-oxopropan-2-yl)-1-(3,4-dihydroxyphenyl)-6,7-dihydroxy-1,2-dihydronaphthalene-2,3-dicarboxylate (compound 1)

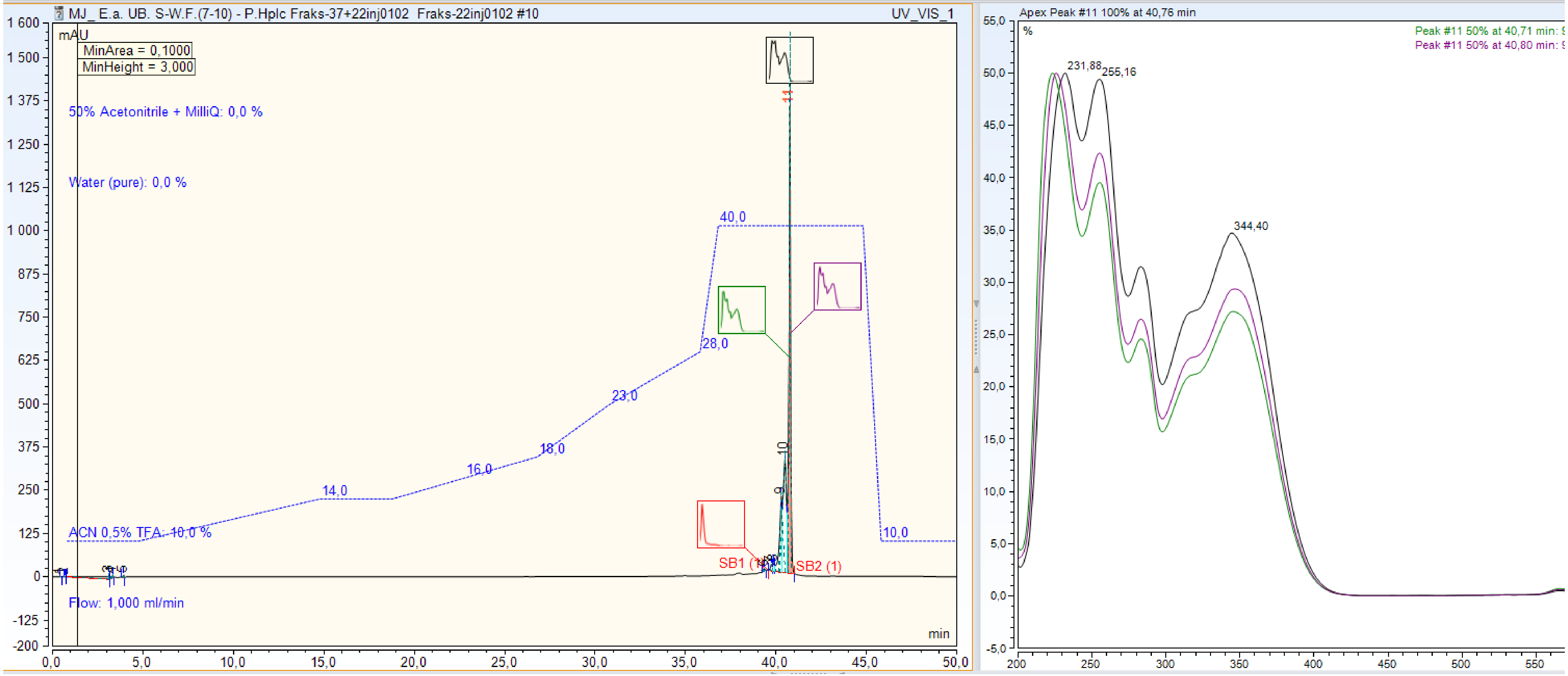

Figure S18. UV spectrum and HPLC chromatogram of of 4-Oxy-(*E*)-caffeoyl-2,3-dihydroxybutanoic acid methyl ester (compound 2). The sample also contains minor amounts of 4-Oxy-(*Z*)-caffeoyl-2,3-dihydroxybutanoic acid methyl ester (compound 3) with Rt 17.80 min.

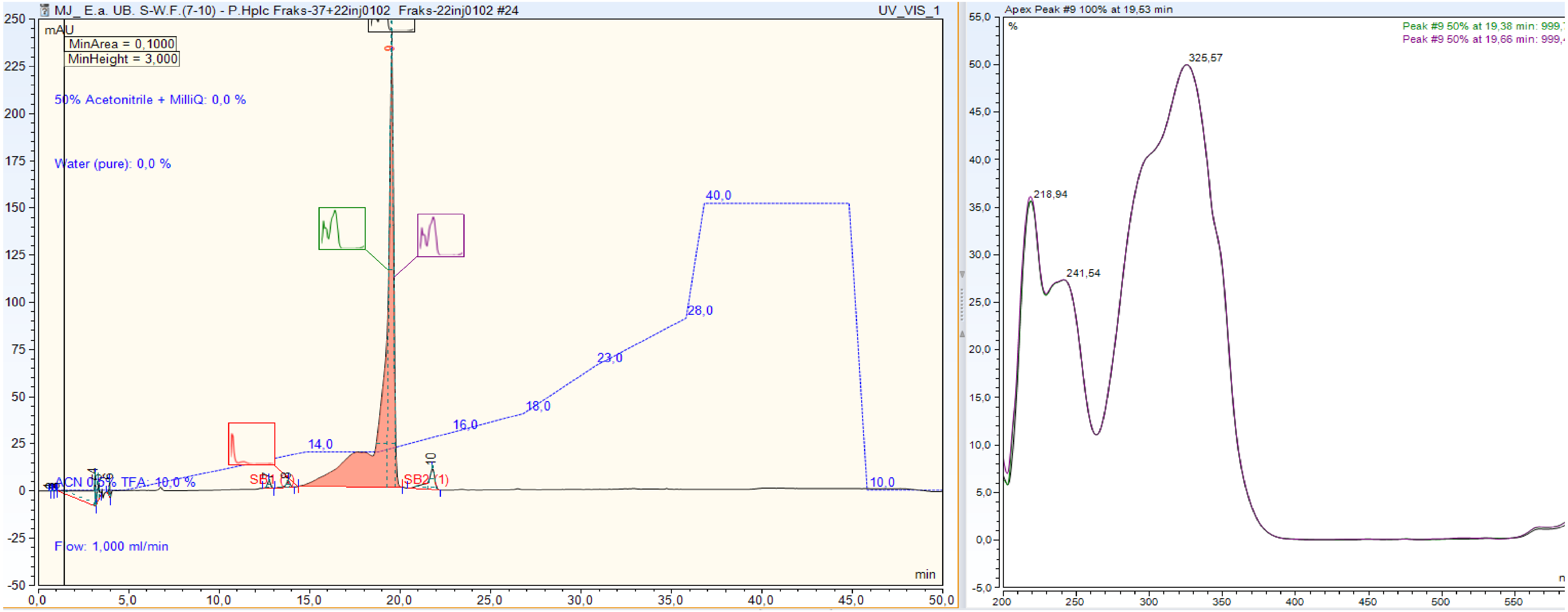

Figure S19. UV spectrum and HPLC chromatogram of rosmarinic acid methyl ester (compound 4)

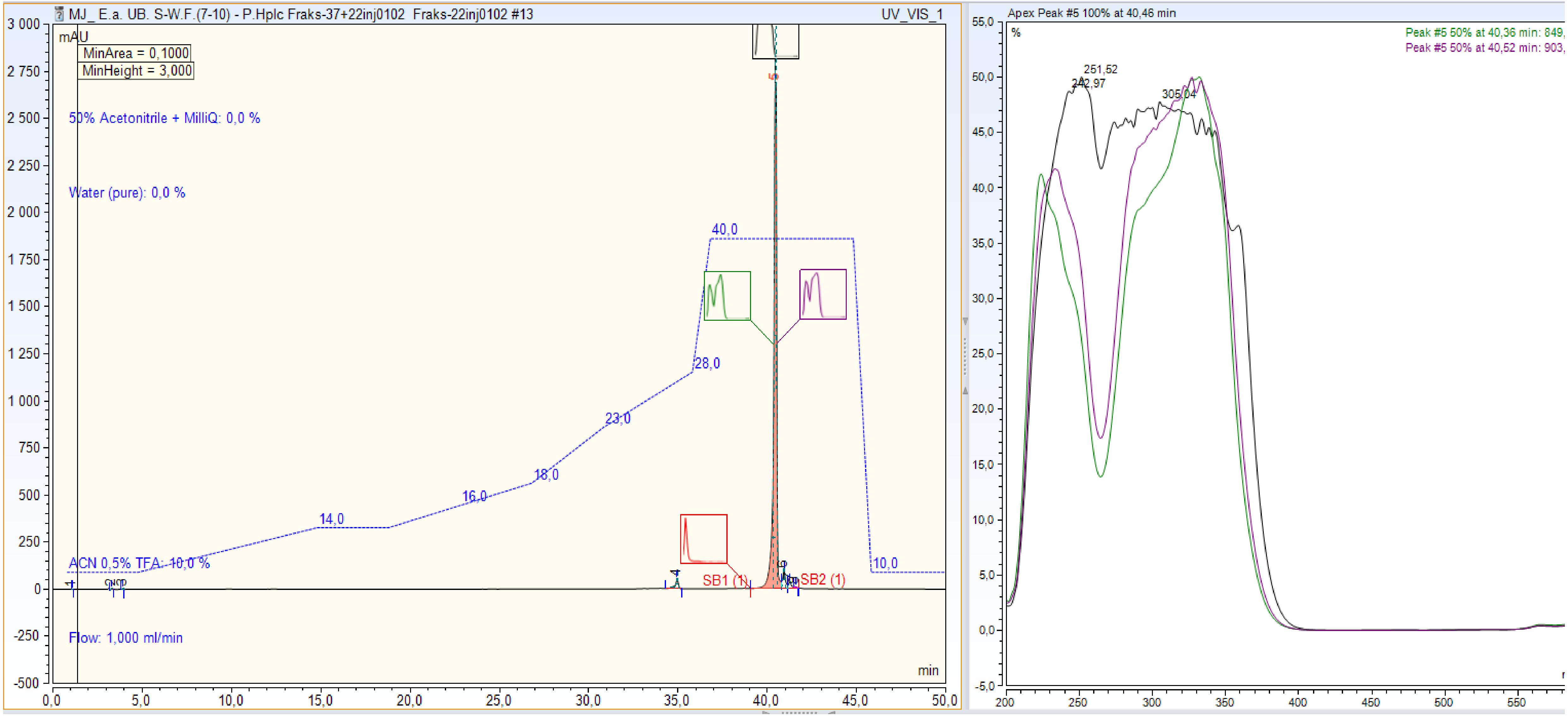

Figure S20. UV spectrum and HPLC chromatogram of (*E*)-caffeic acid (compound 5)

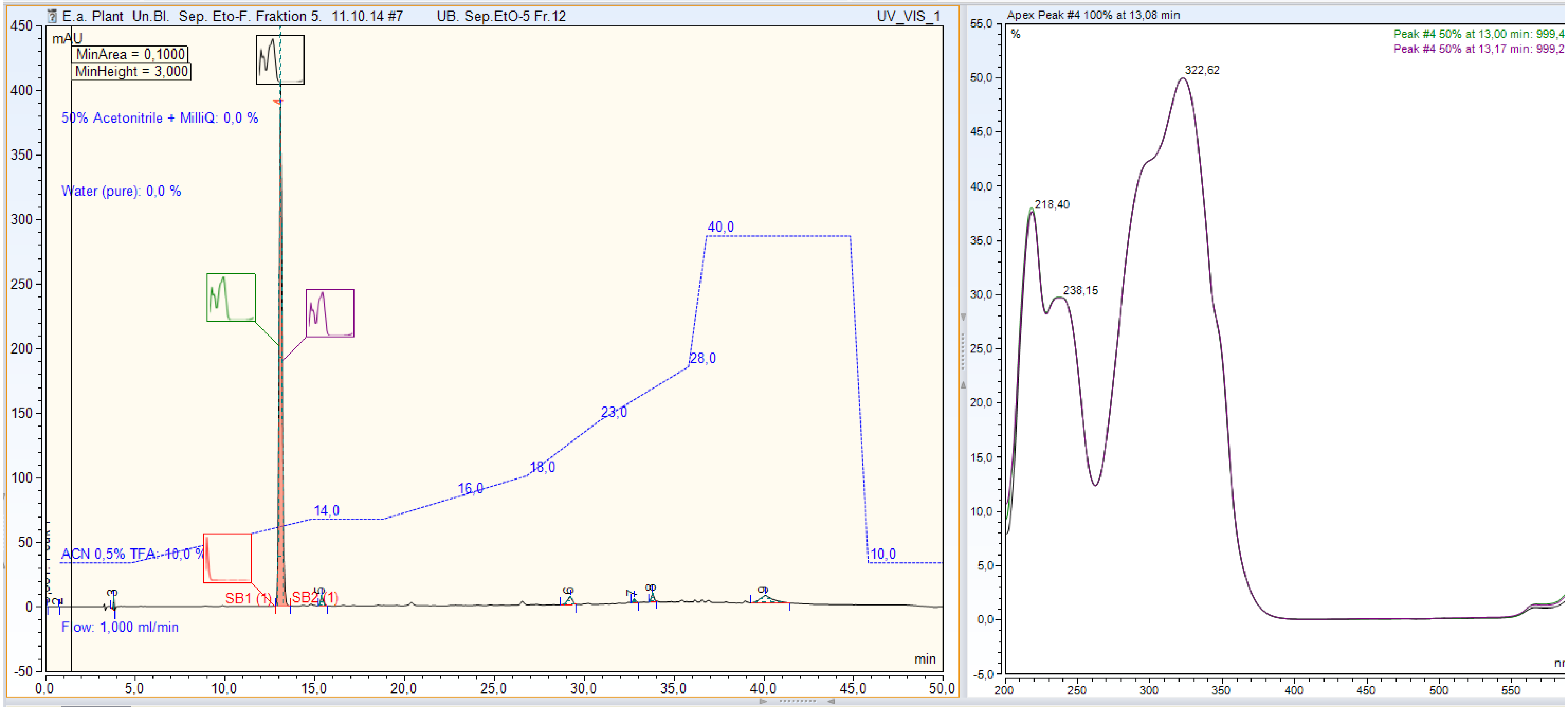

Supplement: Supplementary file 1 [file molecules-28-00385-s001.zip › molecules-2106750-supplementary.pdf]
